# Pathogen-specific host response in critically ill patients with blood stream infections: a nested case–control study

**DOI:** 10.1016/j.ebiom.2025.105799

**Published:** 2025-06-11

**Authors:** Joe M. Butler, Hessel Peters-Sengers, Tom D.Y. Reijnders, Tjitske S.R. van Engelen, Fabrice Uhel, Lonneke A. van Vught, Marcus J. Schultz, Pierre-François Laterre, Bruno François, Miguel Sánchez-García, Eleuterio Lombardo, Timothy E. Sweeney, Marc J. Bonten, W. Joost Wiersinga, Dayle Sampson, Leo C. Bolero, Thomas Yager, Olaf L. Cremer, Brendon P. Scicluna, Tom van der Poll

**Affiliations:** a Centre for Infection and Molecular Medicine, Amsterdam University Medical Centre, Location Academic Medical Centre, University of Amsterdam, Amsterdam, the Netherlands; b Amsterdam Institute for Infection and Immunity, Amsterdam University Medical Centre, Amsterdam, the Netherlands; c Department of Intensive Care Medicine, Amsterdam University Medical Centre, Location Academic Medical Centre, University of Amsterdam, Amsterdam, the Netherlands; d Laboratory of Experimental Intensive Care and Anaesthesiology (L.E.I.C.A), Amsterdam University Medical Centre, Location Academic Medical Centre, University of Amsterdam, Amsterdam, the Netherlands; e Mahidol-Oxford Tropical Medicine Research Unit (MORU), Mahidol University, Bangkok, Thailand; f Nuffield Department of Medicine, University of Oxford, Oxford, UK; g Department of Intensive Care Medicine, Cliniques Universitaires Saint-Luc, Université Catholique de Louvain, UCLouvain, Brussels, Belgium; h Intensive Care Unit and Inserm CIC 1435 & UMR 1092, Limoges University Hospital, Limoges, France; i Critical Care Department, Hospital Clínico San Carlos, Madrid, Spain; j Takeda Madrid, Cell Therapy Technology Centre, Tres Cantos, Spain; k Inflammatix Inc., Sunnyvale, CA, USA; l Department of Medical Microbiology, University Medical Centre Utrecht, Utrecht, the Netherlands; m Julius Centre for Health Sciences and Primary Care, University Medical Centre Utrecht, Utrecht, the Netherlands; n Division of Infectious Diseases, Amsterdam University Medical Centre, Location Academic Medical Centre, University of Amsterdam, Amsterdam, the Netherlands; o Elysium Health, New York, NY, USA; p Al Biostats, South Brisbane, Australia; q Immunexpress, Inc., Seattle, WA, USA; r Department of Intensive Care Medicine, University Medical Centre Utrecht, Utrecht, the Netherlands; s Department of Applied Biomedical Science, Faculty of Health Sciences, Mater Dei Hospital, University of Malta, Msida, Malta

**Keywords:** Bacteraemia, Transcriptome, Host response, Biomarkers, Intensive care

## Abstract

**Background:**

Knowledge of the contribution of the pathogen to the heterogeneity of the host response to infection is limited. We aimed to compare the host response in critically ill patients with a bloodstream infection (BSI).

**Methods:**

RNA profiles were determined in blood obtained between one day before and after a positive blood culture. Differential expression and pathway analyses were performed on independent patients’ samples by RNA sequencing (discovery) or microarray (validation). Additional patients were included for the discovery and validation of transcriptome classifiers of pathogen-specific BSIs. Twenty biomarkers reflecting key host response pathways were measured in blood.

**Findings:**

We included 341 patients, among which 255 with BSI, 25 with viral infection and 61 non-infectious controls. The cultured pathogen explained 41·8% of the blood transcriptomic variance in patients with BSI. Gene set enrichment analysis showed a global resemblance between monomicrobial BSIs caused by *Streptococcus*, *Staphylococcus aureus* and *Escherichia coli*, which were clearly different from BSI caused by coagulase-negative staphylococci or *Enterococcus*. BSI by *Streptococcus* was associated with the highest number of differentially expressed genes, indicating strong innate and adaptive immune activation. An eight-gene streptococcal classifier performed well across different *Streptococcus* species, and was validated in external cohorts. Plasma biomarker profiling showed that *E. coli* BSI was associated with the strongest response in the cytokine and systemic inflammation domain, and *S. aureus* BSI with the strongest endothelial cell activation.

**Interpretation:**

The causative pathogen explains a substantial part of the heterogeneity of the host response in critically ill patients with BSI.

**Funding:**

10.13039/501100006020Center for Translational Molecular Medicine and the 10.13039/501100000780European Commission.

**Research in context**

**Evidence before this study**

The host response to sepsis is highly heterogeneous, which is considered to be related to variations in age, sex (epi)genetic background, comorbidities, chronic medication, and the source of infection. Previous research on the role of the causative pathogen in host response heterogeneity mainly focused on differences between bacterial and viral agents.

**Added value of this study**

We used the Molecular Diagnosis and Risk Stratification of Sepsis (MARS, 2013–2018) prospective cohort to evaluate the host response in critically ill patients with bacterial bloodstream infection (BSI) detected from one day before to three days after admission to the intensive care unit by determining blood transcriptomes and plasma protein biomarkers reflective of key pathophysiological pathways. In BSI patients the cultured pathogen accounted for 41·8% of blood transcriptomic variance; among the five most frequently cultured bacteria *Streptococcus* species caused the most differentially expressed genes relative to non-infected critically ill controls, with gene set enrichment analysis revealing strong activation of both innate and adaptive immune pathways. A diagnostic signature comprising eight genes was developed and shown to effectively distinguish patients with streptococcal BSIs from those without. This signature performed well across various *Streptococcus* species and was validated in external cohorts. Plasma biomarker profiling showed that *Escherichia coli* BSI elicited the strongest response in cytokine and systemic inflammation domains, while *Staphylococcus aureus* BSI induced the most pronounced endothelial cell activation.

**Implications of all the available evidence**

In critically ill patients with BSIs, the causative bacterium accounts for a significant degree of variability in the host response. Rapid identification of the causative pathogen together with the associated immune response can aid clinical decisions not only regarding choice of antimicrobial therapy but also selection of patients for immunotherapy.

## Introduction

Sepsis, defined as a life-threatening organ dysfunction caused by a dysregulated host response to an infection, represents a large global health problem.^1^ A recent Global Burden of Disease study reported 49 million sepsis incident cases and 11 million sepsis-related deaths in 2017, representing nearly 20% of all deaths in that year.^2^ In high-income countries sepsis is the most common cause of in-hospital mortality and associated with high health care costs.^3^^,^^4^ To develop effective treatments, a better understanding of host response heterogeneity in sepsis is essential.

Analyses of the blood leucocyte transcriptome have provided important information about the immune response in patients with sepsis.^5^^,^^6^ These investigations documented that part of the host response heterogeneity in sepsis is due to the causative pathogen, as seen by investigations that compared viral versus bacterial infections.^7^^,^^8^ However, less is known about deviations in the host response due to different bacteria. We recently reported that only a small part of blood transcriptome alterations in patients with sepsis due to community-acquired pneumonia is specific for the causative bacterial pathogen.^9^

Detection of the pathogen in a blood culture (BC) is considered the gold standard for the microbiological diagnosis of sepsis.^10^ We here investigated if a positive BC, drawn from adult patients close to admission to the intensive care unit (ICU), is associated with a pathogen-specific host response. The primary objective was to obtain insight into the contribution of the causative pathogen to the heterogeneity in the immune response to infection in critically ill patients. For this we determined blood transcriptomes and 20 host response biomarkers reflective of key pathophysiological mechanisms implicated in sepsis. BCs are positive in only one third of patients with sepsis, and the long time to positivity hampers its use in the management of sepsis patients in the acute setting.^11^ Therefore, the secondary objective was to identify gene expression classifiers that can assist in the rapid diagnosis of bloodstream infection (BSI) by a specific bacterial pathogen.

## Methods

### Patient selection

We used the Molecular Diagnosis and Risk Stratification of Sepsis (MARS, 2013–2018) prospective cohort of two mixed ICUs in tertiary teaching hospitals (Academic Medical Centre in Amsterdam and University Medical Centre Utrecht)^12^^,^^13^ to identify patients with a BC taken between one day prior to and three days after ICU admission, and from whom a PAXgene tube (Becton–Dickinson) for transcriptomics was obtained between one day before and one day after the BC draw. From this group we identified the patients for whom the BC was positive for bacteria. BSIs were categorized as monobacterial if no other pathogen was detected between two days before and two days after the PAXgene tube draw; if more than one pathogen was detected in this time window the BSI was categorized as mixed. Positive BCs were matched to a clinical episode of infection as assessed by two independent clinicians, who, using all available clinical and microbiological data, also determined that the BC pathogen was causative of the infection, and therefore not a contaminant. A control group of non-infectious (NI) ICU patients was defined as those who were initially suspected of infection for which antibiotics were prescribed and from whom at least one BC was taken, but in whom all BCs and other cultures remained negative, and who eventually were assigned an infection likelihood of none^14^ (hereafter referred to as NI-controls); criteria for infection likelihoods were described in detail.^14^ For all BSIs the source of infection was defined previously.^14^

Patients from either sex were included in the MARS study (ClinicalTrials.gov identifier NCT0195033) using an opt-out method approved by the institutional review boards of both hospitals (IRB No. 10-056C).

### Statistical analyses

Baseline characteristics were compared using ANOVA for normally distributed variables and Kruskal–Wallis test for non-normally distributed variables, determined by visual inspection of histograms and QQ plots. Categorical data were analysed using Fisher's exact test. Survival analysis was conducted using the Kaplan–Meier method and unadjusted Cox proportional hazards models, with only the BSI group as the predictor. We sought to explore drivers of gene expression variation by evaluating seven predictors: cultured microbe (five categories); source of infection (seven categories), Charlson comorbidity index; APACHE IV severity score, age, sex, and RNA-platform (RNAseq, U219 or HTA2·0), see Supplementary Methods for details. There were no missing data observed in any of these variables nor for the survival outcome data.

### Blood transcriptome analyses

Transcriptomes in whole blood were determined by RNA sequencing (seq), U219 microarray (Affymetrix) or GeneChip Human Transcriptome Array (HTA) 2·0 (Thermo-Fisher). For assessment of differential expression of genes and gene pathways we used RNAseq data for discovery and U219 microarray data for validation (different patients), comparing each of the monomicrobial patient groups with NI-controls. We defined a differentially expressed gene (DEG) as a gene with a difference in expression between two groups with a large effect size (Hedges' g > 0·8)^15^ and a concordant direction in the discovery and validation cohort. Note the term ‘effect size’ is used here as a statistical measure of the magnitude of association and does not imply causation, as this is an observational study. Gene set enrichment analysis (GSEA) was performed for each monomicrobial patient group compared to the NI-control group, where ranking of genes was based on Hedges' g. The pathways analysed were taken from the Reactome database (reactome.org),^16^ and which consisted of at least 30 genes. GSEA was performed separately for discovery and validation, from which a combined p value per pathway was calculated using Fisher's method, to which Benjamini-Hochberg correction was applied to adjust for multiple testing. A pathway was defined as significant if the BH-adjusted aggregated p value <0·05 and the direction, as determined by the normalized enrichment score (NES), was concordant between the RNAseq discovery and U219 validation cohorts; non-significant pathways were assigned a zero NES. The association of BSI groups with molecular sepsis endotypes^13^^,^^17^, ^18^, ^19^ was tested using Fisher's exact test. Discovery and validation of transcriptome classifiers specific for a bacterial pathogen were done as described in the Supplementary Appendix.

### Plasma host response biomarkers

In a randomly selected subgroup (n = 150, 72·5%), we measured 20 host response biomarkers in plasma obtained between one day before and one day after the BC draw, as described further in the Supplementary Appendix. We categorized the biomarkers into two pathophysiological domains, “inflammatory and cytokine responses” and “endothelial and coagulation activation”.

### Role of funders

This study was supported by the Centre for Translational Molecular Medicine and the European Commission. The funding sources had no role in the study design, data collection, data analysis, interpretation, or writing of the report.

## Results

### Patient characteristics and outcomes

We identified 255 patients with at least one positive BC between one day prior to and three days after ICU admission (for distribution see Appendix, Table S1), and from whom a PAXgene tube for blood transcriptomics was taken between one day before and one day after the BC draw. Of these, 205 patients had a single pathogen and 50 had multiple pathogens cultured from blood (for details see Appendix, Table S2). Patients with suspected infection who retrospectively were determined to be non-infectious, served as controls (NI-controls; n = 61; Table 1; for admission diagnoses see Appendix, Table S3). We also included patients with viral infection (n = 25; Appendix, Table S2). The final study cohort comprised 341 patients who had their blood transcriptome measured by either RNAseq (n = 132) or microarray (U219, n = 132; HTA2·0, n = 77); for RNA profiling platforms across patient groups see Appendix, Table S4.

**Table 1:** Baseline characteristics and outcome of patients stratified according to BSI groups.

	Coagulase-negative staphylococci	*E.coli*	*Enterococcus*	*S.aureus*	*Streptococcus*	Non-infectious	p value
n	19	40	23	23	41	61	
Demographics							
Age, years, mean (SD)	61·1 (16·1)	62·1 (12·5)	63·2 (11·2)	61·7 (12·1)	60·0 (14·9)	58·4 (17·6)	0·75
Sex, male (n, %)	14 (73·7)	22 (55·0)	11 (47·8)	14 (60·9)	26 (63·4)	39 (63·9)	0·57
Race, non-caucasian (%)	2 (10·5)	1 (2·5)	4 (17·4)	2 (8·7)	2 (4·9)	11 (18·0)	0·11
Body mass index (mean (SD))	27·0 (5·2)	26·6 (6·5)	25·8 (6·4)	26·0 (5·7)	26·0 (5·6)	25·3 (4·8)	0·86
Surgical admission, n (%)	5 (26·3)	8 (20·0)	6 (26·1)	2 (8·7)	5 (12·2)	16 (26·2)	0·33
Chronic comorbidity							
None, n (%)	11 (57·9)	20 (50·0)	6 (26·1)	8 (34·8)	16 (39·0)	32 (52·5)	0·16
Cardiovascular insufficiency, n (%)	1 (5·3)	3 (7·5)	1 (4·3)	2 (8·7)	2 (4·9)	2 (3·3)	0·92
Renal insufficiency, n (%)	2 (10·5)	8 (20·0)	7 (30·4)	5 (21·7)	7 (17·1)	6 (9·8)	0·27
Respiratory insufficiency, n (%)	2 (10·5)	1 (2·5)	3 (13·0)	0 (0·0)	3 (7·3)	4 (6·6)	0·42
Immune deficiency, n (%)	1 (5·3)	10 (25·0)	8 (34·8)	4 (17·4)	7 (17·1)	6 (9·8)	0·055
Malignancy, n (%)	2 (10·5)	10 (25·0)	6 (26·1)	5 (21·7)	10 (24·4)	5 (8·2)	0·14
Chronic obstructive pulmonary disease, n (%)	2 (10·5)	2 (5·0)	5 (21·7)	3 (13·0)	7 (17·1)	6 (9·8)	0·39
Diabetes, n (%)	2 (10·5)	8 (20·0)	4 (17·4)	5 (21·7)	3 (7·3)	13 (21·3)	0·45
Charlson score (median [IQR])	4·0 [2·5, 5·0]	4·0 [3·0, 5·0]	5·0 [4·0, 6·5]	5·0 [4·0, 6·0]	4·0 [3·0, 6·0]	4·0 [2·0, 5·0]	0·068
Source of infection^a^							
Respiratory, n (%)	8 (42·1)	10 (25·0)	6 (26·1)	8 (34·8)	18 (43·9)	na	0·35
Abdominal, n (%)	2 (10·5)	20 (50·0)	13 (56·5)	1 (4·3)	3 (7·3)	na	<0·001
Cardiovascular, n (%)	7 (36·8)	2 (5·0)	3 (13·0)	7 (30·4)	1 (2·4)	na	<0·001
Urinary, n (%)	1 (5·3)	7 (17·5)	6 (26·1)	3 (13·0)	0 (0·0)	na	0·016
Central nervous system, n (%)	1 (5·3)	0 (0·0)	0 (0·0)	3 (13·0)	8 (19·5)	na	0·009
Skin, n (%)	2 (10·5)	5 (12·5)	0 (0·0)	6 (26·1)	8 (19·5)	na	0·11
Unknown, n (%)	5 (26·3)	3 (7·5)	3 (13·0)	3 (13·0)	3 (7·3)	na	0·24
Other, n (%)	2 (10·5)	0 (0·0)	1 (4·3)	7 (30·4)	7 (17·1)	na	0·004
Severity of disease <24 h							
APACHE IV Score, mean (SD)	84·4 (26·9)	105·0 (37·9)	86·1 (22·5)	99·3 (36·7)	81·0 (26·0)	77·3 (36·9)	0·001
Acute physiology score, mean (SD)	72·9 (23·8)	91·4 (35·9)	71·2 (20·0)	85·8 (36·3)	67·9 (25·4)	66·6 (35·3)	0·001
mSOFA score, median [IQR]^b^	7·0 [4·0, 8·0]	9·0 [6·5, 11·5]	8·5 [6·0, 10·8]	9·0 [7·0, 11·0]	7·0 [5·5, 10·0]	6·5 [3·0, 9·0]	0·003
Shock, n (%)^c^	8 (42·1)	28 (70·0)	13 (56·5)	12 (52·2)	20 (48·8)	26 (42·6)	0·13
Therapy <24 h							
Mechanical ventilation (n, %)	14 (73·7)	28 (70·0)	19 (82·6)	18 (78·3)	34 (82·9)	50 (82·0)	0·68
Renal replacement therapy (n, %)	2 (10·5)	3 (7·5)	6 (26·1)	5 (21·7)	2 (4·9)	6 (9·8)	0·085
Outcomes							
ICU length of stay, days, median [IQR]	6·0 [3·0, 12·0]	3·5 [2·0, 11·0]	7·0 [4·0, 9·5]	6·0 [2·5, 13·5]	5·0 [2·0, 10·0]	2·0 [2·0, 4·0]	0·006
Hospital length of stay, days, median [IQR]	37·0 [18·0, 56·0]	18·0 [4·5, 60·0]	15·0 [9·0, 23·5]	28·0 [12·5, 54·0]	16·0 [9·0, 26·0]	11·0 [4·0, 19·0]	0·001
ICU-acquired complications, n (%)^d^							
None	0·7 (0·5)	0·7 (0·5)	0·7 (0·4)	0·7 (0·4)	0·8 (0·4)	0·9 (0·3)	0·29
Acute kidney injury	1 (5·3)	4 (10·0)	2 (8·7)	0 (0·0)	2 (4·9)	6 (9·8)	0·64
Acute respiratory distress syndrome	1 (5·3)	2 (5·0)	1 (4·3)	1 (4·3)	3 (7·3)	1 (1·6)	0·85
ICU-acquired infection	3 (15·8)	6 (15·0)	5 (21·7)	4 (17·4)	5 (12·2)	0 (0·0)	0·031
Mortality							
ICU	4 (21·1)	9 (22·5)	9 (39·1)	5 (21·7)	6 (14·6)	10 (16·4)	0·27
30 days	3 (15·8)	12 (30·0)	13 (56·5)	8 (34·8)	9 (22·0)	11 (18·0)	0·009
90 days	5 (26·3)	15 (37·5)	14 (60·9)	10 (43·5)	11 (26·8)	16 (26·2)	0·041
1 year	9 (47·4)	17 (42·5)	17 (73·9)	15 (65·2)	13 (31·7)	22 (36·1)	0·005

Data presented as mean with standard deviation (SD), or median [interquartile range, IQR], or n (%). Continuous variables reported as mean were compared using the analysis of variance test or if reported as median Kruskal–Wallis rank-sum test was used, resulting in overall p value. Associations between categorical variables were tested using the Fisher's exact test, resulting in overall p value.

na, not applicable, and if true this cell is not included in the calculation of the p value.

a Patients can have multiple sources of infection, and percentages represent the number of each source out of the BSI group sizes.

b mSOFA modified sequential organ failure assessment (excluding central nervous system component). There were 4 missings on mSOFA score (EC = 1; ENT = 1; STR = 2).

c Shock was defined by the use of vasopressors (norepinephrine, epinephrine or dopamine) for hypotension in a norepinephrine-equivalent dose of more than 0·1 μg/kg/min.

d Complications were defined as ICU-acquired when diagnosed more than 48 h after admission to the ICU.

We focused on patient groups with a single bacterial pathogen cultured from blood with a sample size >15 (Table 1). This criteria resulted in five monomicrobial BSI groups: coagulase-negative staphylococci (CoNS, assigned as causative pathogen, i.e., contaminants excluded; n = 19), *Escherichia (E.) coli* (n = 40), *Enterococcus* species (n = 23), *Staphylococcus (S.) aureus* (n = 23), and *Streptococcus* species (n = 41). Pathogens were associated with the source of infection; *E. coli* and *Enterococcus* were especially detected in abdominal and urinary tract infections, *Streptococcus* in respiratory infections, *S. aureus* and CoNS in cardiovascular infections. Mortality differed between groups with *Enterococcus* BSI having the highest mortality (56·5% at day 30); see Appendix, Figure S1 and Table S5.

### Differential gene expression and pathway analysis

**Fig. 1:**
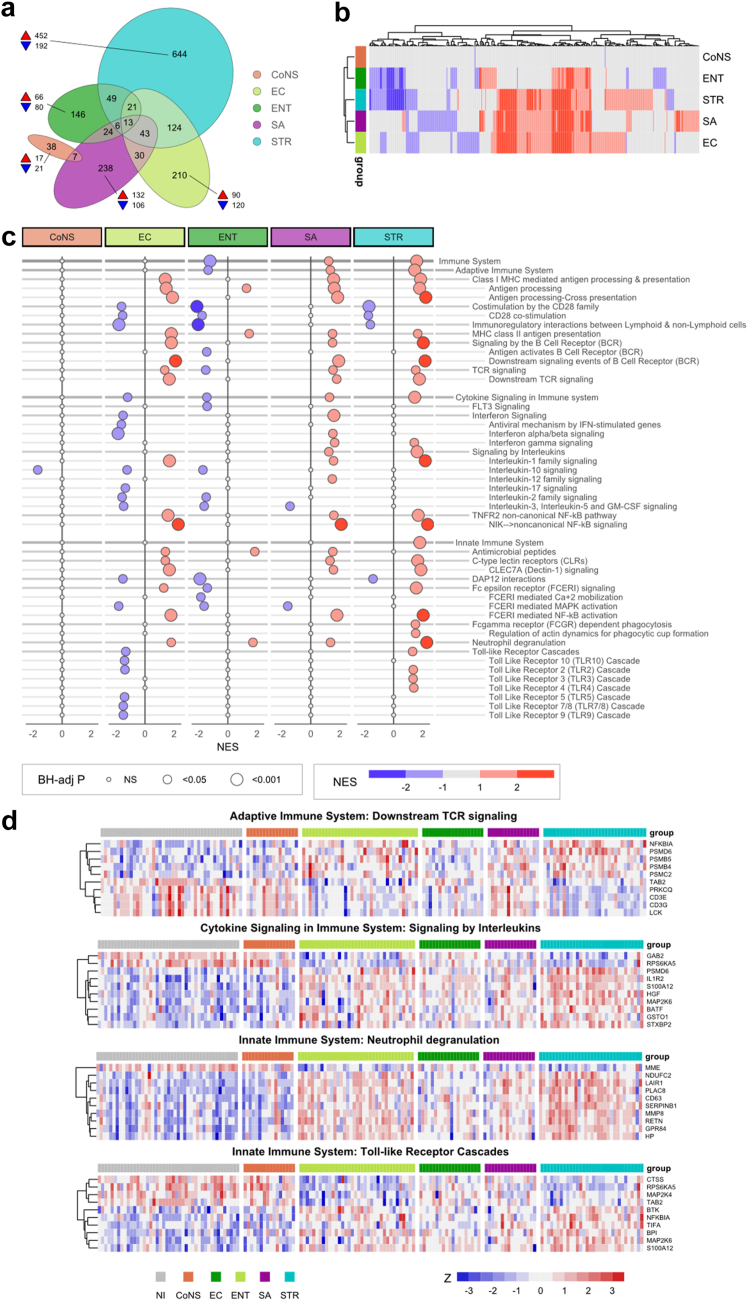
**Patients with different monomicrobial BSIs exhibit distinct blood transcriptomic profiles**. a) Venn–Euler plot showing the number of validated DEGs for each group with a positive blood culture with a single pathogen, compared to non-infectious controls. DEGs were discovered using RNAseq and validated in an independent cohort using U219 microarray; a DEG was defined by having an absolute value of Hedges' g > 0·8 in both cohorts and the direction of regulation being concordant. Not all intersections are shown due to the imperfect solution of the eulerr package; as a consequence 94 DEGs are not depicted. b) Heatmap showing the clustering of BSI groups based on pathway enrichment scores compared to non-infectious controls. Pathways from the four highest levels of the Reactome database that were significant for at least one monomicrobial group (versus non-infectious controls) are shown, i.e., if BH-adjusted-p < 0·05 in both discovery (RNAseq) and validation (U219) cohorts, and the direction of regulation was concordant between them (same sign of normalized enrichment score (NES)). In this way 344 pathways were significant and clustering was based on the mean of the two NES scores from discovery (RNAseq) and validation (U219) cohorts. c) Targeted pathway analysis of immune system pathways. Pathways are shown in a nested manner reflecting the Reactome hierarchical structure. Pathway significance was calculated separately for discovery and validation cohorts; then these two p values were combined using Fisher's method to which BH-adjustment was applied for multiple testing. Pathway direction (up or down regulation) was determined by the mean of two NES values which were calculated separately for discovery and validation. If the pathway direction in the two cohorts were discordant (in opposite direction) the pathway was deemed not significant (NS) and assigned a zero NES. d) Heatmaps showing expression levels of genes in selected pathways from adaptive, innate and cytokine signalling in the immune system. For each gene z-scores were calculated separately for both discovery and validation cohorts. For each pathway the DEGs with the 10 largest F-values (from one-way ANOVA on z-scores are shown, after assessing homogeneity of variance across groups using Levene's test). CoNS, coagulase-negative staphylococci; EC, *E. coli*; ENT, *Enterocococcus*; NI, non-infectious (controls); SA, *S. aureus*; STR, *Streptococcus.*

Our first objective was to identify differences in the blood leucocyte transcriptome response to the five most frequent BSI bacterial pathogens. To this end, we compared RNA profiles of each of the five monomicrobial groups to the NI-control group, using RNAseq (discovery) and U219 arrays (validation) in independent patients (Appendix, Table S6). We defined DEGs as genes that were differentially expressed (and in the same direction) in both the discovery and validation cohorts. Patients with a BC positive for *Streptococcus* exhibited the most DEGs (966), followed by *E. coli* (475), *S. aureus* (436), and *Enterococcus* (291); CoNS BSI was associated with only 51 DEGs (Fig. 1a; Appendix, Figure S2). There were no common DEGs between all five patient groups, but 13 DEGs were shared between *Streptococcus*, *E. coli*, *S. aureus* and *Enterococcus*. The group with the largest response was *Streptococcus* with 644 unique DEGs, which were predominantly up-regulated relative to NI-controls (452).

Untargeted GSEA revealed that patients with *Streptococcus* BSI had the highest number of differentially regulated pathways compared to NI-controls (216 significant pathways), followed by *E. coli* (190), *S. aureus* (191) and *Enterococcus* (129). CoNS had only two significant pathways (both down) compared to NI-controls (Fig. 1b; Appendix, Figure S3). Most pathways in the *Streptococcus* group were up-regulated (154 pathways; with 62 down-regulated). Hierarchical clustering of patient groups by pathway enrichment revealed a degree of global transcriptomic resemblance between *Streptococcus, S. aureus* and *E. coli* groups (Fig. 1b). However, also clear differences were observed between BSI groups with significant pathways unique to each group. Targeting “Immune System” pathways in the Reactome database,^16^ we observed many were upregulated in the *Streptococcus* group (Fig. 1c), including pathways implicated in adaptive immunity (e.g., MHC class II antigen presentation, B cell and T cell signalling), cytokine signalling and innate immunity (e.g., C-type lectin receptor function, Fcγ receptor dependent phagocytosis, neutrophil degranulation and Toll-like receptor cascades), suggesting that *Streptococcus* BSIs are associated with predominantly proinflammatory responses. *E. coli* BSI was accompanied with a mixed pattern of upregulated and downregulated pathways implicated in adaptive and innate immunity, yet with clear downregulation of cytokine signalling and Toll-like receptor cascades. *Enterococcus* BSI was mainly associated with downregulation of immune system related pathways, possibly reflecting an immune suppressed state rendering patients vulnerable to infection by this opportunistic pathogen. Interestingly, CoNS BSI did not induce major gene expression pathways relative to NI-controls, despite careful selection of cases considered genuine infection rather than contaminants. Fig. 1d displays heatmaps of selected immune pathways demonstrating host-response differences between groups at the gene level, with particular contrast between the *Streptococcus* and NI-control groups.

**Fig. 2:**
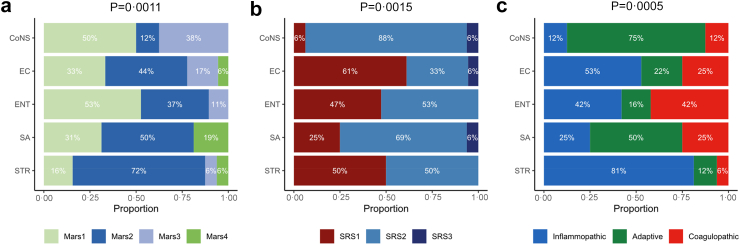
**Association between transcriptomic endotypes and BSI group**. (a) Distribution of the four MARS endotypes, (b) the three SRS endotypes, and (c) the Inflammopathic, Adaptive, and Coagulopathic endotypes for each of the five BSI groups, assessed by combining the RNAseq and U219 cohorts. Fisher's exact test was used to test for association between BSI group and endotype. p values indicate the probability of observing the data or something more extreme assuming the null hypothesis of no association between BSI group and endotype is true. CoNS, coagulase-negative staphylococci; EC, *E. coli*; ENT, *Enterocococcus*; SA, *S. aureus*; STR, *Streptococcus.*

Recently, patients with sepsis have been separated in subtypes based on blood transcriptomes, including Mars1 to Mars4,^13^ sepsis response signature (SRS)1 to SRS3,^17^^,^^18^ and subtypes named “inflammopathic”, “adaptive” and “coagulopathic”.^19^ BSI pathogens significantly associated with these molecular sepsis subtypes (Fig. 2). *Streptococcus* BSI particularly associated with the Mars2 and inflammatopathic subtypes, which both are characterized by increased expression of genes related to innate immune activation. *S. aureus* and CoNS BSI especially associated with the SRS2 and adaptive subtypes, which both relate to enhanced expression of genes involved in adaptive immune activation. The distribution of other pathogens across molecular sepsis subtypes was less consistent. These data suggest that, in bacteraemic ICU patients, molecular subtypes are partially driven by the pathogen, but that other–not microbe related–factors contribute as well.

### Transcriptomic classifier discovery and validation

We next wished to determine whether we could identify transcriptome classifiers of BSI caused by each of the five pathogens evaluated above. For this, we used RNA expression data derived from three different gene expression platforms, i.e., RNAseq, U219 microarray, and HTA2·0 microarray, of which the first two platforms were also used to generate the results shown in Figs. 1 and 2 (Appendix, Table S4).

**Fig. 3:**
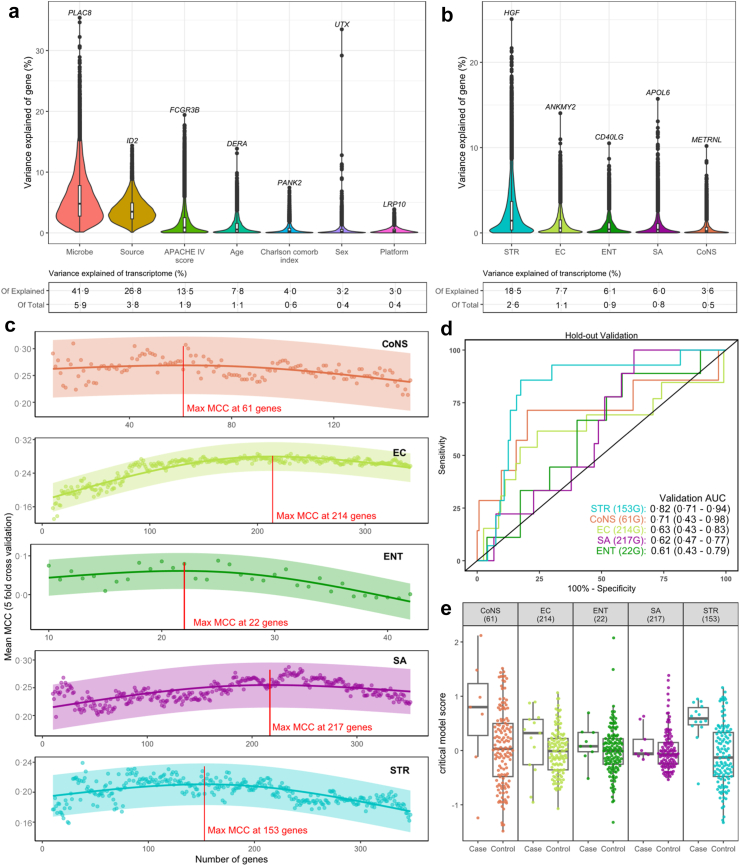
Discovery and validation of transcriptomic classifiers for BSIs. a) Variance partitioning (in the monomicrobial BSI cohort) revealed that the cultured microbe explained the most transcriptomic variance compared to other clinical variables. Violin plots show the distribution of percentages of the variance explained by each explanatory variable over all 13,294 genes (protein coding common between three platforms); the gene which explained the most by each explanatory variable is labelled. Boxplots inside the violin plots show the median gene value (black bar) and interquartile range (white box). Genes shown in black circles are outliers, defined as points falling more than 1·5 times the interquartile range (IQR) from the first or third quartile. The table below shows the overall transcriptomic variance explained, both as a fraction of total transcriptomic variance including residual variance, and as a fraction of the summed explained transcriptomic variance excluding residual variance. b) Variance partitioning focussed on pathogen by dichotomizing the cultured microbe variable into indicator variables, one for each monomicrobial group (non-infectious control group as baseline). Note that the regression model for this analysis included all the same baseline characteristics and platform as in panel A, but only the microbe variables are shown. This analysis indicates that *Streptococcus* infections explain the most transcriptomic variance. Boxplots inside the violin plots show the median gene value (black bar) and interquartile range (white box), Genes shown in black circles are outliers, defined as points falling more than 1·5 times the interquartile range (IQR) from the first or third quartile. c) Gene set size selection using ULTRA with repeated cross-validation within the discovery cohort and Matthews correlation coefficient (MCC) to identify the optimal classification performance. The gene set with the maximum MCC was selected as the critical set size. After repeated cross-validation a mean value for each set size was calculated (shown as dots), then a natural spline model was fit to these data points to determine the overall maximum MCC, shown by the vertical red line. The natural spline model was fitted with 3 internal knots placed in the default positions (at the 25th, 50th, and 75th percentiles of the data). The shaded band is a 95% confidence interval of the spline model. d) Receiver operating curves (ROCs) showing critical model classification performance in the hold-out validation cohort for each of the BSI groups. Number of genes in each classifier is shown in parentheses prior to the letter “G”. The 95% CIs of the AUCs, estimated by DeLong's method, are shown in the second parentheses. After the critical set-size was determined, this number of genes was used to build the critical model using the discovery data, this final critical model was then assessed in the hold-out validation. Note that this entire analysis was performed on the full BC-taken patient cohort which included viral and polymicrobial infections as controls. e) Distribution of critical model scores, used for the final classification, in the hold-out validation for each of the BSI groups. CoNS, coagulase-negative staphylococci; EC, *E. coli*; ENT, *Enterocococcus*; SA, *S. aureus*; STR, *Streptococcus.*

First, we examined our approach of normalizing and combining data from the three gene expression platforms by performing a variance partitioning analysis^20^; this showed that the platform explained a low amount of transcriptomic variance in comparison with clinical variables (Fig. 3a), providing validity to our co-normalization approach. Strikingly, the cultured microbe explained the most overall transcriptional variance (41·8% of the summed explained transcriptomic variance excluding residual variance, or 5·9% of total variance), more than the source of infection (26·8% and 3·8% respectively) and clinical variables such as severity of disease, age, comorbidities, and sex. Splitting the multi-categorical microbe variable into its five monomicrobial groups showed that *Streptococcus* explained the most transcriptomic variance (18·5% and 2·6% respectively; Fig. 3b).

Next, we sought to discover and validate transcriptional classifiers for each pathogen group. For this cases and controls for each target pathogen were divided into a discovery (60%) and validation (40%) cohort (Appendix, Tables S7–S11). Here, in order to emulate the real-life ICU population in which a BC would be taken, the control group included patients who had a BC taken and had positive microbiology for either fungal BSI or virus infection, or positive cultures for other or mixed BSIs (except those including the target pathogen). We applied a univariate logistic tandem regression algorithm (ULTRA) to the discovery data (see Appendix for details) to observe an inverted-U shape relationship between the number of genes used and the classifier performance for each of the target BSIs (Fig. 3c). This shape reflects the signal-to-noise principle underlying the ULTRA approach wherein genes are added in order of their significance such that with smaller set-sizes the genes are more likely to represent true signal, whereas with excessively high set-sizes (including genes lower down the ranked list) genes are more likely to represent noise. From the inverted-U we identified the gene set-size that maximized the classification performance and assessed the classifier with this optimal set-size in the validation data (Fig. 3d). We found a 153-gene classifier for *Streptococcus* with good performance (validation area under the curve (AUC) of 0·82). The classifier for CoNS contained 61 genes with a validation AUC of 0·71. Transcriptome classifiers for *Enterococcus*, *S. aureus* and *E. coli* BSIs did not perform well in independent patients of the validation cohort (AUC ≤0·65). The distribution of the classifier scores between cases and controls in the validation cohorts is shown in Fig. 3e.

**Fig. 4:**
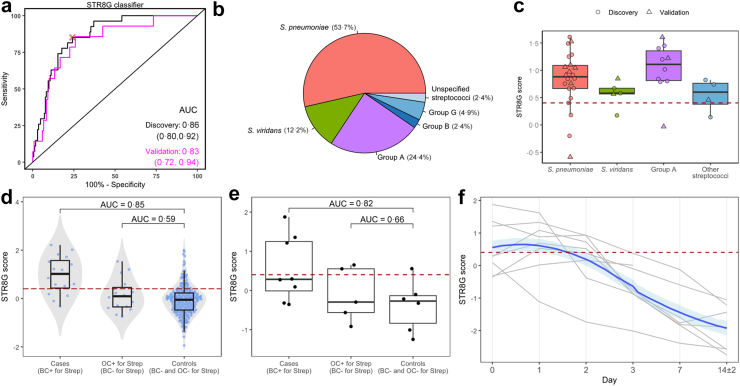
Performance of the STR8G *Streptococcus* classifier. a) ROC plot showing STR8G classifier performance in the discovery (black) and hold-out validation (magenta) cohorts. The 95% CIs of the AUCs, estimated by DeLong's method, are shown in parentheses. The red “X” represents the optimal threshold for the STR8G classifier as defined by the Youden index in the discovery cohort. b) Pie chart showing breakdown of *Streptococcus* BSIs into species subgroups, including the Lancefield groupings (groups A, B and G), in the combined discovery and validation cohort. c) Boxplots showing the STR8G classifier score across *Streptococcus* species subgroups. The “Other streptococci” group included group B, group G and unspecified streptococci. The red dashed line represents the optimal threshold defined by the Youden index in the discovery cohort. d) STR8G classifier performance assessed in an external cohort of patients with community-acquired pneumonia admitted to the ward (not ICU). The red dashed line represents the optimal threshold defined by the Youden index in the original discovery cohort. OC: other cultures (i.e., other than blood culture). e) STR8G classifier performance assessed in an external cohort of patients with sepsis due to severe community-acquired pneumonia in the ICU (SEPCELL study). This data was taken from a randomized controlled trial and performance of STR8G is shown here for whole blood transcriptome samples (RNAseq) taken within 18 h of ICU admission (day 0), prior the initiation of intervention or placebo. The red dashed line represents the optimal threshold defined by the Youden index in the original discovery cohort. OC: other cultures (i.e., other than blood culture). f) Estimation of the STR8G classifier trajectory over time in the cases (BC + for *Streptococcus*) of the SEPCELL external cohort. The blue line depicts the mean trajectory over time with a 95% CI of the mean (shaded band). This was calculated using LOESS regression to estimate a trajectory per patient with corresponding standard errors, from which a random value was generated to calculate a mean value across patients; this was repeated 1000 times to generate the overall trajectory, and the 95% CI calculated from the 2·5th and 97·5th percentiles. The grey lines represent individual patients. The red dashed line represents the optimal threshold defined by the Youden index in the original discovery cohort.

Finally, we sought to identify a minimal gene set classifier for the 153-gene *Streptococcus* signature. From the 153 genes we iteratively selected the top up- and down-regulated genes based on AUC; in each iteration genes with a high correlation (Pearson's *r,* |*r*|> 0·7) with the selected genes were removed and from the remaining set the next two top genes were added to the model until no further improvement in model performance was observed. In this way an eight-gene classifier (STR8G) was identified (Appendix, Figure S4a). The AUC of this STR8G classifier was 0·86 in the discovery cohort and 0·83 in the validation cohort (Fig. 4a; Appendix, Figure S4b and c). The expression of each of the eight classifier genes in the discovery and validation cohorts is shown in the Appendix, Figure S4d and e. We also investigated how the classifier performed in the subset of patients who had their blood for transcriptomics drawn on the exact same day as the BC draw and observed a slight improvement in both discovery (AUC of 0·88) and validation (AUC of 0·85) cohorts (Appendix, Figure S5). Further details of the STR8G including gene coefficients and confusion matrix metrics at the optimal threshold can be found in the Appendix, Table S12.

### Further evaluation of the STR8G *Streptococcus* classifier

Next we sought to evaluate the performance of the STR8G classifier across different *Streptococcus* species. 22 of 41 (53·7%) of *Streptococcus* BSI were due to *S. pneumoniae* (Appendix, Table S2; Fig. 4B), with group A streptococci and *S. viridans* also contributing substantially (24·4% and 12·2% respectively). The other *Streptococcus* groups (B, G and unspecified) were low in numbers and combined into one “other streptococci” group. The STR8G classifier performance was stable across all four *Streptococcus* groups (Fig. 4c).

We further investigated the performance of the STR8G classifier in an independent cohort of 274 patients with community-acquired pneumonia who were admitted to a general hospital ward (i.e., not critically ill).^21^ Of these, 16 patients had a positive BC for *Streptococcus* on admission (15 *S. pneumoniae*, one *S. pyogenes*), and an additional 16 patients had other (non-BC) cultures positive for *Streptococcus* (15 *S. pneumoniae*, one *S. pyogenes*) but with negative BCs. The STR8G classifier performed well for the BC positive cases compared to the controls (n = 242) with an AUC of 0·85 (Fig. 4d). In contrast, the STR8G classifier did not discriminate between patients with non-BC culture-positive streptococcal infection and controls (AUC of 0·59).

Finally, we investigated the performance of the STR8G classifier in sepsis patients with community-acquired pneumonia enrolled in a randomized controlled clinical trial testing mesenchymal stem cells.^22^ RNAseq was performed at admission and various timepoints in the patient's ICU stay. We identified 20 patients in which a BC was taken, of whom eight had a positive BC for *Streptococcus* (seven *S. pneumoniae*, one *S. pyogenes*), five with other (non-BC) cultures positive for *S. pneumoniae* and seven controls (all cultures negative for *Streptococcus*). In agreement with the ward cohort, the STR8G classifier performed well for BC positive cases (AUC of 0·82), but not in patients who only had *Streptococcus* identified in a non-BC (AUC of 0·66; Fig. 4e). In BC positive cases the average STR8G score increased during the first day of ICU admission, then fell below the threshold within the second day (Fig. 4f).

### Host response biomarkers

**Fig. 5:**
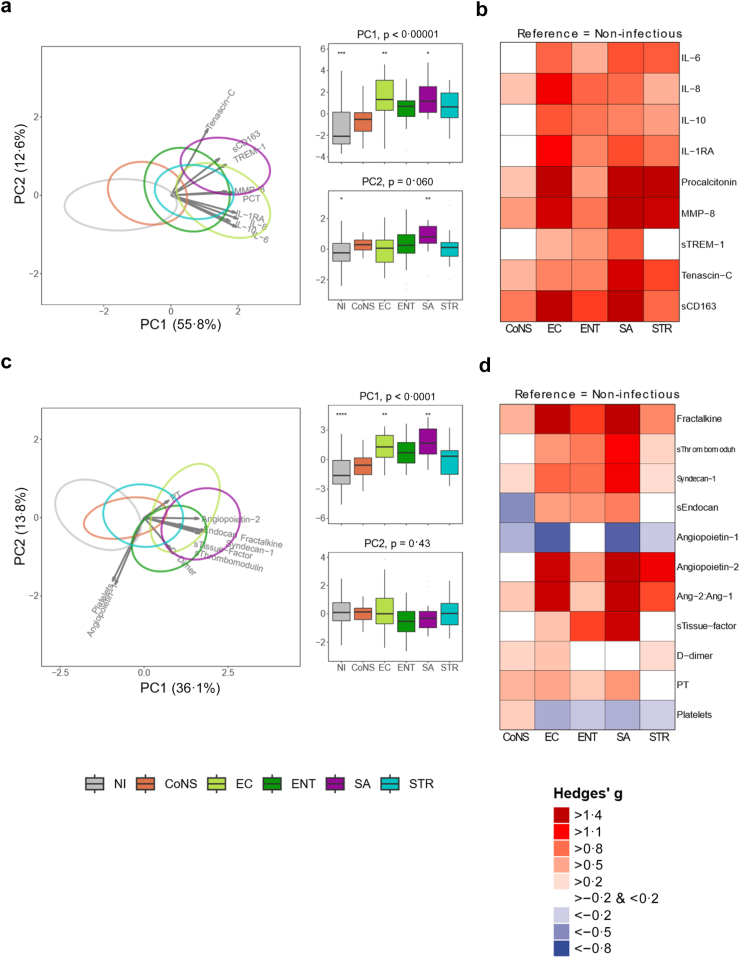
**Plasma biomarker host response profiles**. Comparison of plasma biomarkers indicative of “Cytokine release and systemic inflammatory responses” (panels a and b) and “Endothelial cell and procoagulant responses” (panels c and d), in patients with bacteraemia stratified according to the causative pathogen. Data are presented as principal component analysis (PCA) plots (panels a and c, left). Ellipses in PCA plots represent patient data points for each BSI group (not shown here for clarity). Arrows in PCA plots indicate direction and extent of correlation of plasma markers with loadings of PCA components. The boxplots (panels a and c, right) show the difference in the first (PC1) and second component (PC2) loadings between groups (asterisks indicate differences between groups compared to the overall-mean of the PC; ∗p < 0·05, ∗∗p < 0·01, ∗∗∗p < 0·001, ∗∗∗∗p < 0·0001). Exact p values for 5a PC1 (NI, p = 0·0002; EC, p = 0·0083; SA, p = 0·018), PC2 (NI p = 0·031; SA, p = 0·0043). Exact p values for 5c PC1 (NI, p < 0·0001; EC, p = 0·0064; SA, p = 0·0024). For heatmaps (panels b and d) each group was compared to non-infectious controls and Hedges' g was used to evaluate the difference in plasma levels between these two groups. Ang, angiopoietin; CD, cluster of differentiation; IL, interleukin; MMP, matrix metalloproteinase; PT, prothrombin time; RA, receptor antagonist; s, soluble; TREM, triggering receptor expressed on myeloid cells; CoNS, coagulase-negative staphylococci; EC, *E. coli*; ENT, *Enterocococcus*; SA, *S. aureus*; STR, *Streptococcus*.

To obtain further insight into pathogen-specific host response aberrations in bacteraemic patients on the ICU we measured 20 biomarkers indicative of the pathophysiological domains “inflammatory and cytokine responses” and “endothelial and coagulation activation” in the five monomicrobial BSI groups: CoNS (n = 15), *E. coli* (n = 25), *Enterococcus* species (n = 16), *S. aureus* (n = 15), *Streptococcus* species (n = 26), and NI-controls (n = 52) (Fig. 5). Further details are provided in the Appendix (Figure S6, Table S13). While particularly *E. coli*, *Streptococcus*, *S. aureus* and *Enterococcus* elicited strong and overlapping biomarker responses that were clearly different from those in NI-controls, differences were observed across pathogens. Generally, *E. coli* elicited the strongest responses in the cytokine release and systemic inflammation domains, while *S. aureus* induced the strongest changes in biomarkers reflecting endothelial activation and function.

## Discussion

We report the blood transcriptional response in critically ill patients with BSIs stratified according to the five most commonly cultured bacteria. Variance partitioning analysis, which estimates how much variance in a response is explained by a certain factor while adjusting for all other factors,^20^ indicated that the causative pathogen explained most of the transcriptional variance out of all explanatory variables considered, including severity of disease and source of infection, with the method of analysis (RNAseq versus microarray) only having a small impact. Immune pathway enrichment analysis demonstrated overlapping changes in the blood transcriptome associated with BSIs caused by *Streptocococcus*, *S. aureus* and *E. coli*, characterized by increased expression of genes implicated in both innate and adaptive immune responses. Streptococcal BSI was accompanied by the strongest transcriptomic changes and an eight-gene signature could identify *Streptococcus* in BCs with a high accuracy across different streptococcal species, cohorts, and care settings. These results indicate that the blood transcriptome can provide concurrent information about the causative pathogen and the associated immune response in patients with BSIs.

Heterogeneity has been implicated as an important factor in the high number of negative trials in sepsis. In an attempt to reduce heterogeneity distinct sepsis phenotypes have been identified based on clustering analyses of blood transcriptome data^6^^,^^13^^,^^17^, ^18^, ^19^ or clinical and biomarker data.^6^^,^^23^ Different sepsis phenotypes may respond differently to immunomodulatory interventions,^23^, ^24^, ^25^ paving the way for predictive enrichment of future trials. In this context, the hyperinflammatory phenotype originally described in patients with acute respiratory distress syndrome has also been found in sepsis, particularly in patients with bacteraemia,^23^^,^^26^ aligning with the strong proinflammatory immune activation in BSIs caused by virulent pathogens like *Streptococcus*, *S. aureus* and *E. coli* in this study. We in addition show that the distribution of transcriptome phenotypes^13^^,^^17^, ^18^, ^19^ differs across BSIs in a pathogen-specific manner. Collectively, these results support the notion that the causative pathogen explains a substantial portion of the host response heterogeneity in BSIs.

Enterococcal BSI was associated with the highest mortality rates. Notably, it is uncertain whether enterococcal BSI is driving mortality or merely a marker of morbidity associated with higher risk of dying and/or conditions that render patients more vulnerable to enterococcal infection.

Previous investigations reported on differences in blood transcriptomes of patients with documented bacterial and viral infections.^7^^,^^8^ Our study focused on bacterial BSIs and comparison between bacteria cultured from blood; we included patients with viral infections (diagnosed by PCR from different specimens, not necessarily blood) merely as a control group for the classifier analysis to emulate the real life situation in the ICU.

The STR8G classifier performed well across different *Streptococcus* species, which may be related to common immunogenic components and virulence factors, including lipoteichoic acid, peptidoglycan and cholesterol-dependent cytolysins such as pneumolysin in *S. pneumoniae* and streptolysin in *S. pyogenes*.^27^ Quickly detecting and identifying a causative pathogen in BSIs allows for prompt initiation of appropriate antibiotic therapy, which has been linked to lower morbidity and mortality rates.^28^ The advent of culture-independent techniques offers potential solutions to the challenges faced in the microbiological diagnosis of BSIs.^29^ However, these methodologies can also detect microorganisms whose relevance to the patient's condition is not clear. Concomitant assessment of the host response offers an opportunity to determine whether the detected pathogen exists in the context of an immunological state consistent with infection, such as recently indicated by metagenomic next-generation sequencing of whole blood and plasma samples of critically ill patients.^28^ In this context, the STR8G signature identifies not only patients with a high likelihood of streptococcal bacteraemia, but also signifies a subgroup with strong immune system activation, which may aid decisions on antimicrobial and immunomodulatory therapies. The fact that the STR8G classifier performed well when *Streptococcus* was cultured from blood, but not in patients who had *Streptococcus* identified from a non-BC, suggests that this RNA signature at least in part is induced by direct contact of blood leukocytes with the pathogen in the circulation.

Plasma protein biomarker profiling indicated that BSIs caused by *E. coli* were associated with the most profound activation within the cytokine and inflammation domain, which is in agreement with the strong activation of innate immune pathways in blood leukocytes. *S. aureus* was most potent in triggering procoagulant and endothelial activation, which may be related to its expression of certain virulence factors, like α-toxin and coagulases, and its capacity to activate the complement system, which is closely interconnected with the coagulation system.^30^

Our study has strengths and limitations. Because we strove to define pure mono-microbial BSIs, the CoNS, *Enterococcus*, and *S. aureus* groups were relatively small. This strict definition ensured that each case group truly represented a single pathogen with the aim of reducing noise (and increase the effect-size), but with the cost of a smaller sample size. Although the STR8G classifier was validated in external cohorts across different care settings throughout Europe, the 95% CIs of the validation AUCs and other measures of diagnostic accuracy were wider than those in the discovery cohort. Thus further validation in studies with larger sample sizes is required before the implications and importance of the STR8G classifier in clinical practice can be established. We defined genes as differentially transcribed only if changed in a concordant way in independent discovery and validation cohorts, analysed with different RNA expression platforms, providing robustness to the results. Yet, this data were from two Dutch ICUs, and may not be generalizable to other countries. Our analysis focused on gene-level expression changes and did not account for alternative splicing patterns, which can produce functionally distinct mRNA isoforms from the same gene locus, representing an exciting avenue for future deeper mechanistic investigations. A dedicated team judged whether a cultured microorganism was causative of an infection; yet, the possibility of contamination is not totally excluded. Our study did not take within genus/species variation (e.g., certain virulence factors) in BSI pathogens into account and does not elucidate which microbial components drive leucocyte responses. While a future PCR based test can provide rapid results, costs may be high for use in routine clinical practice. The plasma biomarker panel was targeted and limited to 20 parameters, providing only information about selected pathophysiological pathways. For pathway and protein analyses, confounders were intentionally excluded to provide real-world estimates for each BSI group. Consequently, these analyses do not allow for conclusions regarding the independent contribution of BSI groups to pathways and biomarkers.

Sepsis triggers a highly diverse host response, influenced by factors such as age, sex (epi)genetic makeup, pre-existing health conditions, ongoing medications, and the source of infection. Previous studies exploring the impact of the causative pathogen on host response variability reported varying results related to small sample sizes and different culture sources.^31^ We here show that the causative bacterium significantly influences the host response variability in critically ill patients with BSIs. Swift identification of the responsible pathogen and its associated immune response can inform clinical decisions, including selection of appropriate antimicrobial therapy and identifying patients who are most likely to benefit from immunotherapy.

## Contributors

JMB, HPS, and TvdP conceived and designed the study. TDYR and TSRvE performed laboratory analyses. JMB and HPS performed data analysis and verified the underlying data of the study. LAvV, MJS, PFL, BF, MSG, EL, MJB, WJW, and OLC were essential for patient recruitment and critically reviewed the manuscript. FU, TES, DS, LCB, TY, and BPS significantly contributed to data analysis and interpretation. JMB, HPS and TvdP wrote the first version of the manuscript. All authors contributed revisions, edits, and approved the manuscript for submission. All authors had full access to all the data in the study and accept responsibility for the decision to submit for publication.

## Data sharing statement

Data are available upon reasonable request on approval of a written request to TvdP (Amsterdam UMC, University of Amsterdam). Array data are available at the Gene Expression Omnibus public repository of NCBI under accession number GSE65682 (U219) and GSE134346 (HTA2·0). RNA sequencing data are available at the Gene Expression Omnibus public repository of NCBI under accession number GSE276095.

## Declaration of interests

Dr. H. Peters-Sengers was supported by the Dutch Kidney Foundation (Nierstichting) postdoc KOLFF grant 19OK009. Dr. van Engelen was supported by an Amsterdam UMC PhD scholarship grant. Dr. van Vught was supported by the *Netherlands Organisation* for *Health Research* and *Development* ZonMW VENI grant 09150161910033. Dr. Schultz is a former research coordinator at Hamilton Medical AG; this role was unrelated to the research presented. Dr Francois received consulting fees from Enlivex and Inotrem, and support for travel from Eagle. Dr. Lombardo is a Takeda employee and owns stock in the company; he received payments from ESAME and Francisco de Votoria University for presentations at Master of Advanced Therapies, and from iBET for accommodation and travel support to attend a thesis defence. Dr Sweeney is an employee of, and stockholder in Inflammatix, Inc. Dr. Bonten is CEO of the European Clinical Research Alliance on Infectious Diseases (Ecraid); in this role he received grants from Sequiris, TechnoPhage, Attea Pharma, Phaxiam and Janssen Vaccines (all paid to Eucraid). Marc Bonten also received grants from Merck, GSK, European Commission, and consulting fees from Merck, GSK and Janssen Vaccines (all paid to UMC Utrecht). Dr. Wiersinga reports grants from the Netherlands Organisation for Health Research and Development (ZonMw), EU/Eurostars and Moderna outside the submitted work. Drs. Simpson and Bolero disclose that they were former employees and shareholders of Immunexpress Inc. Dr. Yager declares that he owns shares of stock and has been granted stock options in Immunexpress Inc; he is a current employee of Immunexpress, Inc. Dr. Cremer received grants from the Centre of Translational Medicine, Health Holland, ZonMW, EU Digital Europe, Presymptom Health Ltd (all paid to UMC Utrecht), he received payment for his participation in the Committee for European Education in Anaesthesiology (paid to UMC Utrecht). Dr. Cremer is Chair of the Science & Innovation Committee of the Dutch Society of Intensive Care, member of the External Project Advisory Committee of the BEATsep Consortium and member of the Scientific Advisory Board of the International Clinical research centre Brno (all without payment). Dr. van der Poll reports grants from Immunexpress, EU/Horizon 2020 (FAIR, Immunosep), the Ministry of Economic Affairs & Health Holland, and the Dutch Thrombosis Foundation, as well as a consultancy with Matisse (all paid to the institution); he is a member of Data Safety Monitoring Board of REMAP-CAP (no payment). Drs. Butler, Reijnders, Uhel, Laterre, Sanchez and Scicluna have no competing interests to declare.

## Appendix A Supplementary data

Supplementary Appendix Final V2
