# Supplementary material for: Pathogen-specific host response in critically ill patients with blood stream infections: a nested case–control study
Source: eBioMedicine. 2025 Jun 11;117:105799. doi: 10.1016/j.ebiom.2025.105799 (PMC12182767; doi:10.1016/j.ebiom.2025.105799)
Supplement: Supplementary Appendix Final V2 [file mmc1.docx]

**Supplementary appendix**

**Pathogen-specific host response in critically ill patients with blood stream infections**

**Contents**

[Supplementary Methods – Definitions, Biomarker assays, Bioinformatics 2](#_Toc190875962)

[Table S1: Timing of blood culture draw relative to ICU admission 5](#_Toc190875963)

[Table S2: Blood stream infection (BSI) pathogens 6](#_Toc190875964)

[Table S3: Admission diagnoses of non-infectious control patients 8](#_Toc190875965)

[Table S4: Transcriptome platforms used for RNA profiling across patient groups 9](#_Toc190875966)

[Table S5: Hazard ratio’s for 30-day and 1-year mortality 10](#_Toc190875967)

[Table S6: Transcriptomic classifier discovery and validation cohorts 11](#_Toc190875968)

[Table S7. Baseline characteristics and outcome of patients stratified for the CoNS classifier analysis 12](#_Toc190875969)

[Table S8. Baseline characteristics and outcome of patients stratified for the *E. coli* classifier analysis 13](#_Toc190875970)

[Table S9. Baseline characteristics and outcome of patients stratified for the *Enterococcus* classifier analysis 14](#_Toc190875971)

[Table S10. Baseline characteristics and outcome of patients stratified for the *S. aureus* classifier analysis 15](#_Toc190875972)

[Table S11. Baseline characteristics and outcome of patients stratified for the *Streptococcus* classifier analysis 16](#_Toc190875973)

[Table S12: Additional details and performance statistics of the STR8G classifier 17](#_Toc190875974)

[Table S13: Plasma host response biomarker pairwise comparisons (unadjusted) 18](#_Toc190875975)

[Figure S1: Kaplan-Meier Survival Curves for BSI groups 21](#_Toc190875976)

[Figure S2: Scatter plots showing the effect size of gene expression when comparing each bacterial group to NI-controls for all genes in two independent cohorts; discovery cohort (RNAseq) versus validation cohort (U219) 22](#_Toc190875977)

[Figure S3: Heatmaps showing mean normalized enrichment scores (NES) from GSEA pathway analyses comparing each BSI group to non-infectious controls. 23](#_Toc190875978)

[Figure S4: Selection and performance of an eight-gene classifier for *Streptococcus* BSIs (STR8G) 24](#_Toc190875979)

[Figure S5: STR8G classifier performance evaluated in patients with BC taken on same day as PAXgene blood draw. 25](#_Toc190875980)

[Figure S6: Plasma host response biomarkers stratified by BSI group 26](#_Toc190875981)

[References 27](#_Toc190875982)

# Supplementary Methods – Definitions, Biomarker assays, Bioinformatics

**Comorbidities**

Cardiovascular compromise was defined as a medical history of congestive heart failure, chronic cardiovascular disease, myocardial infarction, peripheral vascular disease or cerebrovascular disease. Malignancy was defined as a medical history of either metastatic or not metastatic solid tumor, or hemodynamic malignancy. Renal insufficiency was defined as a history of chronic renal insufficiency, or treatment with chronic intermittent hemodialysis or continuous ambulatory peritoneal dialysis. Respiratory insufficiency was defined as a history of chronic respiratory insufficiency, chronic obstructive pulmonary disease, or treatment at home with oxygen or ventilator support. Immune compromise was defined as a history of immune deficiency, human immunodeficiency virus (HIV) infection, acquired immune deficiency syndrome (AIDS), asplenia, or chronic use of corticosteroids, antineoplastic or other immune suppressive medications. Chronic comorbid conditions were scored using the Charlson comorbidity index^1^.

**Organ dysfunctions**

Shock was defined by the use of vasopressors (norepinephrine, epinephrine or dopamine) for hypotension in a norepinephrine-equivalent dose of more than 0.1 µg/kg/min. Acute respiratory distress syndrome (ARDS) was prospectively defined using strict preset criteria^2^.

**Intensive care unit (ICU)-acquired complications**

ICU-acquired infection was defined as any new-onset infection starting more than 48 hours after ICU admittance, and for which the attending physician started a new antibiotic regimen. ICU-acquired ARDS was defined as ARDS diagnosed more than 48 hours after ICU admission. Acute renal injury (AKI) was prospectively defined according to RIFLE and KDIGO criteria^34^.

**Mortality outcome**

For calculation of 30-day, 90-day, and 1-year mortality, the death date from the Dutch national registration of personal details (Dutch BRP) was used.

**RNA isolation and profiling**

Total RNA was isolated by means of the QIAcube machine (Qiagen, Venlo, the Netherlands) in combination with the Blood mRNA kit (Qiagen) according to manufacturer’s procedures. RNA quality was assessed by bioanalysis (Agilent, Santa Clara, CA), with all samples having RNA integrity numbers above six. Total RNA concentrations were determined by Qubit® 2·0 Fluorometer (Life Technologies, Carlsbad, CA, USA). For RNA sequencing (RNAseq) libraries were prepared by means of the KAPA RNA HyperPrep with RiboErase (Roche) as per the manufacturer’s instructions. Libraries were sequenced using the Illumina HiSeq4000 (Illumina, San Diego, CA, USA) to generate 50bp single-end reads. The sequence read quality was assessed using FastQC methods (version 0·11·5; Babraham Institute, Babraham, Cambridgeshire, UK). Trimmomatic version 0·39 ^5^ was used to trim the Illumina adapters and filter low quality reads and ambiguous nucleotide-containing sequences. Low quality leading (three nucleotides) and trailing (three nucleotides) bases were removed from each read. A sliding window trimming using a window of four and a phred score threshold of 15 nucleotides was used to access the quality of the reads. After pre-processing, the remaining high-quality reads were aligned against the human Genome Reference Consortium Build 38 (GRCh38, Ensembl 84) using Hisat2 (2·2·0) ^6^ with default parameters. Count data were generated by means of the HTSeq method ^7^. Independent RNA samples (i.e., from other patients used for validation) were measured by U219 microarray (Affymetrix) or the GeneChip Human Transcriptome Array (HTA) 2·0 (Thermo-Fisher) as previously described ^8^ ^9^.

**Variance partitioning**

For every gene we evaluated the variance of expression explained by each predictor in a linear model with seven predictors: cultured microbe (five categories); source of infection (seven categories: abdominal, cardiovascular, respiratory, skin, urinary, mixed and other); Charlson comorbidity index; APACHE IV score; age; sex; and platform (RNAseq, U219 or HTA2·0). In the secondary analysis we used indicator variables for the individual cultured microbes. For both analyses the NI-control group was the baseline group.

The variance explained by predictor *p* of the total transcriptomic variance (% of *TTV_p_*) was calculated as:

${\% of TTV}_{p}=\frac{\sum_{g}^{G} {ESS}_{p,g}}{\sum_{g}^{G} {TSS}_{g}}$ where ${TSS}_{g}=\sum_{p}^{P} {ESS}_{p,g}+{RSS}_{g}$

where *TSS_g_* is the total sum of squares for gene *g* (*g* ϵ G), *ESS_p,g_* is the explained sum of squares by explanatory variable *p* for gene *g*, and *RSS_g_* is the residual sum of squares for gene *g*. We also calculated the variance explained by predictor *p* of the summed explained transcriptomic variance (% of SETV*_p_*), summed over all predictors (but excluding residual variance):

${\% of SETV}_{p}=\frac{\sum_{g}^{G} {ESS}_{p,g}}{\sum_{g}^{G} {ESS}_{g}}$ where ${ESS}_{g}=\sum_{p}^{P} {ESS}_{p,g}$

**Transcriptomic classifier discovery and validation**

For discovering transcriptomic classifiers specific for each of the five bacterial pathogens we expanded the control group with the following patient groups: fungal BSI, other BSIs, mixed BSIs, viral infections, and NI-controls. This was done to emulate a real-life ICU population in which a BC could grow a variety of different pathogen classes. We here included additional patients in whom the blood transcriptome was measured by HTA2·0 microarray, as well as those measured in the pathway analyses (RNAseq, U219). Discovery and validation cohorts were constructed using a 60:40 temporal split. Specifically, for every target pathogen, cases were ordered by the time of ICU admission from which we identified the date at which 60% of cases had an admission before that date (defining the discovery cases) and 40% after that date (defining the validation cases). The gene expression matrices from the three platforms (RNAseq, U219 or HTA2·0) were separately quantile normalized and then z-score standardized (centred and scaled) before being merged (by HGNC gene symbol) into a single meta expression matrix. For the microarrays the most significant probe per gene was selected to represent that gene. Low expressed genes were then removed by using a coefficient of determination threshold of 4 in the RNAseq cohort (the most sensitive platform for measuring low expressed genes), leaving a final meta expression matrix containing 10,893 genes.

For every target pathogen to be classified we defined three groups: cases, controls and exclusions, wherein cases were patients with a BC positive for a single “target” pathogen, exclusions patients with a positive BC for a target pathogen plus another pathogen, and controls all patients remaining. To help overcome the problem of model selection when the number of features (k) is much higher than the number of observations (n) we developed an algorithm named the univariate logistic tandem regression algorithm (ULTRA). This is inspired by the independence assumption of the naïve Bayes classifier in that each feature/predictor is assumed to be independent from all other predictors. In brief, a standard univariate logistic regression was performed separately for every gene; thus for gene *g*:

$$p\left( case \right)=\frac{1}{1+exp(-\left( \alpha+\beta_{g}z_{g} \right))}$$

where *z_g_* is the vector of z-scaled expression values, and *α* (intercept) and *ß_g_* are the parameters to be estimated. This was performed for all genes in G, and then the genes were ordered by their univariate area under the receiver operating characteristic curve (AUROC). Next, based on this ordered list of genes, we performed an analysis to determine a critical number of genes *c_max_* which can be interpreted as the set which maximizes the signal to noise ratio. Let *C* represent the set of the *c* most discriminative genes (highest univariate AUROC), then for each patient we estimated the mean of the $\beta_{g}z_{g,i}$ values over these *c* genes, let this be denoted $\bar{\beta z}_{i}$ for patient *i*:

$$\bar{\beta z}_{i}=\left( \sum_{g in C} \beta_{g}z_{g,i} \right)/c$$

Then for each gene set size *c* we calculated the ULTRA “multivariate” AUROC (note this is not the same as multivariate regression in which multiple coefficients are estimated simultaneously) using the $\bar{\beta z}_{i}$ as the continuous diagnostic score, over the *c* genes.

1. Identifying the critical number of genes using ULTRA with repeated cross-validation

ULTRA was performed with repeated cross-validation to provide a robust Mathhews Correlation Coefficient (MCC) estimate. On the discovery data we implemented a 50% train and a 50% test split: the 50% train set (i.e. 30% of total data) was used to estimate the univariate coefficients and also a Youden index for every set size from 1 to 1000. Then each model was validated in the test data using MCC to quantify classification performance. The model that maximized this MCC metric, containing *c_max_* genes, was then fit (i.e. univariate coefficients estimated) using the entire 60% of discovery data, and its performance was assessed in the hold-out (40%) validation data using AUROC. If the AUROC exceeded 0·8 in the hold-out validation set we sought to find a minimal gene set with similar performance to the critical set.

1. Identifying a minimal set of genes using ULTRA for Streptococcus (STR8G classifier)

For any target pathogen with an AUC greater than 0·8 in the hold-out validation, we sought to find a minimal set of informative genes by removing highly correlated genes (i.e. genes with redundant information) from the critical set. First, we broke down the critical gene set into two sets: up-regulated and down-regulated genes. Both these gene sets were ranked by univariate AUROC. The top up-regulated gene and down-regulated gene were selected, call this the (provisional) minimal set. Then any gene from the up or down gene sets with a high correlation (Pearson’s correlation coefficient greater than 0·7) with any gene in the minimal set was removed. From each of the remaining up and down gene sets, the gene with the next highest univariate AUROC was then added to the minimal set. This process was iterated until the goodness of fit (binomial deviance) was not significantly improved by adding the two additional genes, as determined by a likelihood ratio test. Note to calculate the binomial deviance a further univariate logistic regression was performed where the continuous diagnostic score $\bar{\beta z}_{i}$ was the single predictor, also known as Platt scaling.

The optimal number of genes was deduced by iterating a likelihood ratio test comparing a nested model (n genes) to a more complex model (n+2 genes) until the more complex model was no longer a significantly better fit than the nested model. Once the optimal number of genes was identified the optimal threshold was selected using the Youden index cutoff point which maximizes sensitivity and specificity.

This ULTRA method has been developed as a R package and is available on github ( <https://joemb1.github.io/ultra/> ).

For the eight selected genes, we tested the linearity assumption of the logistic regression model using component-plus-residual plots and found that all eight met this assumption.

To further test the performance of the *Streptococcus* STR8G classifier we selected four additional independent groups of ICU patients from the MARS cohort; these patients were not in the discovery or validation cohorts described above: (1) patients in whom all cultures (including BCs) were negative; (2) patients in whom a culture (other than BC) was positive for *Streptococcus*, yet with negative BCs; (3) patients with a BC positive for *Streptococcus* and another bacterium (mixed BSI; this was the exclusion group from our main analysis above). Additional validation was done in patients admitted to the general ward with community-acquired pneumonia included in the ELDER-BIOME observational study (clinicaltrials.gov identifier NCT02928367)^10^. For estimation of the temporal trajectory of the STR8G classifier we used sequential samples (ICU admission, days 1, 2, 3, 7 and 14±2) from patients enrolled in a randomized controlled clinical trial testing mesenchymal stem cells (Cx611) in patients with severe community-acquired pneumonia (Cx611-0204 SEPCELL Study; EUDRA-CT 002994-39)^11^. Estimation of the average trajectory was calculated using LOESS regression to estimate a trajectory per patient with corresponding standard errors, from which a random value was generated to calculate a mean value across patients; this was repeated 1000 times to generate the overall trajectory and CI.

**Host response biomarkers**

We measured 17 host response biomarkers on a BioPlex-machine (Bio-Plex 200, Bio-Rad, Hercules, CA, USA) using a custom-made Luminex multiplex assay (R&D Systems Inc, Minneapolis, MN, USA) as described ^12^. Biomarkers were categorized into two pathophysiological domains: interleukin (IL)-6, IL-8, IL-10, and IL-1 receptor antagonist (IL-1RA), matrix metalloproteinase (MMP)-8, soluble triggering receptor expressed on myeloid cells (sTREM)-1, soluble cluster of differentiation (sCD)163, tenascin-C and procalcitonin (PCT) (reflecting inflammatory and cytokine responses); fractalkine, syndecan-1, endocan, soluble thrombomodulin, angiopoietin-1, and angiopoietin-2, soluble tissue factor and D-dimer (reflecting endothelial and coagulation activation). Prothrombin time and platelet counts were determined as part of regular patient care. Differences in individual biomarker levels between groups were quantified using Hedges’ g^13^ and visualised using heatmaps. Normality was tested using visual inspection of QQ plots, all of which were met, and homogeneity of variance with a Levene’s test and if not me a robust sandwich estimator (HC3) was applied.

# Table S1: Timing of blood culture draw relative to ICU admission

| **Day relative to ICU admission** | **Frequency (%)** |
| --- | --- |
| -1 | 29 (11.4) |
| 0 | 190 (74.5) |
| 1 | 26 (10.2) |
| 2 | 7 (2.7) |
| 3 | 3 (1.2) |

# Table S2: Blood stream infection (BSI) pathogens

**Monomicrobial BSIs (N=205)**

| **Species** | **N (%)** |
| --- | --- |
| **Coagulase-negative staphylococci**  *Staphylococcus epidermidis*  Coagulase-negative staphylococci (unspecified)  *Staphylococcus capitis*  *Staphylococcus haemolytic*  *Staphylococcus hominis* | **19 (9.3)**  10  6  1  1  1 |
| ***Escherichia coli*** | **40 (19.5)** |
| ***Enterococcus***  *Enterococcus faecium*  *Enterococcus faecalis*  *Enterococcus* species (unspecified) | **23**  15  5  3 |
| ***Staphylococcus aureus*** | **23 (11.2)** |
| ***Streptococcus***  *Streptococcus pneumoniae*  Group A  *Streptococcus viridans*  Group G  Group B  *Streptococcus* species (unspecified) | **41**  22  10  5  2  1  1 |
| **Other**  *Klebsiella pneumoniae*  *Pseudomonas aeruginosa*  *Bacteroides fragilis*  *Candida albicans*  Gram-negative bacillus (not specified)  *Bacillus species*  *Citrobacter koseri*  *Clostridium* species  *Enterobacter cloacae*  *Proteus mirabilis*  *Bacteroides ovatus*  *Bacteroides thetaiotaomicron*  *Bacteroides uniformis*  *Clostridium bifermentans*  *Clostridium perfringens*  *Cryptococcus neoformans*  *Enterobacter aerogenes*  *Enterobacter asburiae*  *Enterobacter cloacae* complex  *Enterobacter* species  *Haemophilus influenzae*  *Klebsiella oxytoca*  *Listeria monocytogenes*  *Pantoea agglomerans*  *Propionibacterium* species  *Pseudoclavibacter* species  *Salmonella* group B  *Serratia marcescens* | **59 (28.8)**  12  10  3  3  3  2  2  2  2  2  1  1  1  1  1  1  1  1  1  1  1  1  1  1  1  1  1  1 |

**Mixed BSIs (N=50)**

| **Species** | **N (%)** |
| --- | --- |
| **Mixed Gram-negative**  *Citrobacter koseri, Klebsiella oxytoca*  *Enterobacter cloacae, Klebsiella pneumoniae*  *Escherichia coli, Klebsiella pneumoniae*  *Escherichia coli, Proteus mirabilis*  *Escherichia coli, Pseudomonas aeruginosa* | **5 (10.0)**  1  1  1  1  1 |
| **Mixed Gram-positive**  Coagulase-negative staphylococci*, Enterococcus* species  *Enterococcus faecium, Staphylococcus epidermidis*  Coagulase-negative staphylococci*, Staphylococcus aureus*  *Enterococcus faecium, Lactobacillus species*  *Enterococcus faecium, Staphylococcus* species  *Enterococcus faecalis, Staphylococcus epidermidis*  *Enterococcus faecalis, Staphylococcus haemolytic*  *Enterococcus faecium, Staphylococcus haemolytic*  *Staphylococcus aureus, Streptococcus dysgalactiae*  *Staphylococcus aureus, Staphylococcus epidermidis*  *Staphylococcus aureus, Streptococcus* group B *(S· agalactiae)*  *Staphylococcus aureus, Streptococcus pneumoniae*  *Staphylococcus epidermidis, Streptococcus anginosus*  *Staphylococcus hominis, Streptococcus pneumoniae*  *Staphylococcus schleiferi, Streptococcus* β-haemolytic | **19 (38.0)**  3  2  2  1  1  1  1  1  1  1  1  1  1  1  1 |
| **Mixed other**  *Escherichia coli, Staphylococcus epidermidis*  *Abiotrophia species, Fusobacterium necrophorum*  Anaerobe gram-negative bacillus (unspecified), coagulase-negative staphylococci, *Streptococcus mitis, Streptococcus pneumoniae*  Anaerobe gram-negative bacillus (unspecified)*, Staphylococcus hominis*  *Bacillus* species*, Enterobacter cloacae, Escherichia coli*  *Bacteroides thetaiotaomicron, Enterococcus faecium*  Coagulase-negative staphylococci*, Escherichia coli*  *Eggerthella lenta, Escherichia coli*  *Enterobacter aerogenes, Enterococcus avium, Escherichia coli*  *Enterobacter asburiae, Enterococcus faecium*  *Enterobacter cloacae, Enterococcus faecium, Escherichia coli, Klebsiella pneumoniae*  *Enterococcus faecium, Escherichia coli*  *Enterococcus faecium, Enterococcus faecalis, Pseudomonas aeruginosa*  *Enterococcus faecium, Enterococcus* species*, Escherichia coli*  *Enterococcus species, Escherichia coli, Klebsiella oxytoca*  *Enterococcus species, Escherichia coli, Klebsiella pneumoniae, Streptococcus* β-haemolytic  *Enterococcus species, Klebsiella pneumoniae*  *Escherichia coli,* Gram-positive bacillus (not specified)  *Escherichia coli, Staphylococcus hominis*  *Klebsiella pneumoniae, Staphylococcus epidermidis*  *Micrococcus luteus, Pseudomonas aeruginosa*  *Morganella morganii, Proteus mirabilis, Streptococcus haemolyticus* group G  *Neisseria meningitidis, Staphylococcus simulans*  *Proteus mirabilis, Staphylococcus epidermidis*  *Pseudomonas aeruginosa, Staphylococcus epidermidis, Staphylococcus hominis* | **26 (52.0)**  2  1  1  1  1  1  1  1  1  1  1  1  1  1  1  1  1  1  1  1  1  1  1  1  1 |

**Viruses (N=25)**

| Influenza  Coronavirus  Metapneumovirus  Cytomegalovirus  Parainfluenza  Influenza and Rhinovirus  Respiratory syncytial virus  Respiratory syncytial virus and Rhinovirus | **25**  12  3  3  2  2  1  1  1 |
| --- | --- |

# Table S3: Admission diagnoses of non-infectious control patients

|  | **Number (%)** |
| --- | --- |
| Respiratory failure | 13 (21.3) |
| Acute neurological disease (cerebrovascular accident, haemorrhage) | 10 (16.4) |
| Cardiac arrest | 8 (13.1) |
| Cardiac failure | 5 (8.2) |
| Gastrointestinal surgery | 4 (6.6) |
| Trauma | 3 (4.9) |
| Unspecified | 3 (4.9) |
| Cardiac surgery | 2 (3.3) |
| Diabetic ketoacidosis | 2 (3.3) |
| Dissecting aortic aneurysm | 2 (3.3) |
| Acid-base electrolyte disturbance | 1 (1.6) |
| Complications after kidney transplantation | 1 (1.6) |
| Drugs overdose | 1 (1.6) |
| Endarterctomy | 1 (1.6) |
| Gastrointestinal haemorrhage | 1 (1.6) |
| Hepatic failure | 1 (1.6) |
| Near drowning | 1 (1.6) |
| Pulmonary embolism | 1 (1.6) |
| Retroperitoneal lymph node dissection | 1 (1.6) |
|  | **61** |

# Table S4: Transcriptome platforms used for RNA profiling across patient groups

| **Group** | **RNAseq** | **U219** | **HTA2·0** | **Total** |
| --- | --- | --- | --- | --- |
| CoNS | 11 (8.3) | 5 (3.8) | 3 (3.9) | 19 (5.6) |
| *E· coli* | 14 (10.6) | 22 (16.7) | 4 (5.2) | 40 (11.7) |
| *Enterococcus* | 4 (3.0) | 15 (11.4) | 4 (5.2) | 23 (6.7) |
| *S· aureus* | 8 (6.1) | 8 (6.1) | 7 (9.1) | 23 (6.7) |
| *Streptococcus* | 18 (13.6) | 14 (10.6) | 9 (11.7) | 41 (12.0) |
| Other | 20 (15.2) | 22 (16.7) | 16 (20.8) | 58 (17.0) |
| Mixed | 19 (14.4) | 20 (15.2) | 12 (15.6) | 51 (15.0) |
| Virus | 17 (12.9) | 3 (2.3) | 5 (6.5) | 25 (7.3) |
| NI-control | 21 (15.9) | 23 (17.4) | 17 (22.1) | 61 (17.9) |

Note: RNAseq (discovery) and U219 (validation) were used for differential gene expression and pathway analyses (Fig. 1, Fig. 2, and supplementary Fig. S1 and Fig. S2). For evaluation of pathogen-specific classifiers for CoNS, *E· coli*, *Enterococcus*, *S· aureus* and *Streptococcus* data from all three platforms (RNAseq, U219 and HTA2·0) were combined and a 60:40 temporal split was used for discovery and validation respectively (Fig. 3, Fig. 4 and supplementary Fig S3).

# Table S5: Hazard ratio’s for 30-day and 1-year mortality

| **30-days** | **Hazard Ratio** | **Lower 95%CI** | **Upper 95%CI** | **p-value** |
| --- | --- | --- | --- | --- |
| NI-control | 1.00 (ref) |  |  |  |
| CoNS | 0.86 | 0.24 | 3.08 | 0.82 |
| *E. coli* | 1.80 | 0.79 | 4.07 | 0.16 |
| *Enterococuccus* | 3.47 | 1.55 | 7.75 | 0.002 |
| *S. aureus* | 2.05 | 0.82 | 5.10 | 0.12 |
| *Streptococcus* | 1.16 | 0.48 | 2.81 | 0.74 |
|  |  |  |  |  |
| **One-year** | **Hazard Ratio** | **Lower 95%CI** | **Upper 95%CI** | **p-value** |
| NI-control | 1.00 (ref) |  |  |  |
| CoNS | 1.32 | 0.61 | 2.87 | 0.48 |
| *E. coli* | 1.31 | 0.69 | 2.46 | 0.41 |
| *Enterococuccus* | 2.80 | 1.48 | 5.30 | 0.001 |
| *S. aureus* | 2.15 | 1.11 | 4.15 | 0.022 |
| *Streptococcus* | 0.86 | 0.43 | 1.70 | 0.66 |

# Table S6: Transcriptomic classifier discovery and validation cohorts

| **Pathogen** | **Discovery** | | **Validation** | |
| --- | --- | --- | --- | --- |
|  | **Cases** | **Controls** | **Cases** | **Controls** |
| CoNS | 12 (3.8) | 160 (51.1) | 7 (2.2) | 134 (42.8) |
| *E· coli* | 27 (8.4) | 166 (51.6) | 13 (4.0) | 116 (36.0) |
| *Enterococcus* | 14 (4.4) | 171 (53.3) | 9 (2.8) | 127 (39.6) |
| *S· aureus* | 14 (4.5) | 171 (54.6) | 9 (2.9) | 119 (38.0) |
| *Streptococcus* | 27 (8.2) | 170 (51.4) | 14 (4.2) | 120 (36.3) |

#

The control group for each pathogen consisted of all BSIs except for those growing the pathogen that defined the respective cases (including fungal and mixed BSIs), documented viral infections and NI-controls. Discovery and validation cohorts were constructed using a 60:40 temporal split.

# Table S7. Baseline characteristics and outcome of patients stratified for the CoNS classifier analysis

|  | Discovery | | Validation | |
| --- | --- | --- | --- | --- |
|  | Case | Control | Case | Control |
| n | 12 | 160 | 7 | 134 |
| BSI group |  |  |  |  |
| CoNS | 12 (100) | 0 (0·0) | 7 (100) | 0 (0·0) |
| *E. coli* | 0 (0) | 20 (12·5) | 0 (0) | 20 (14·9) |
| *Enterococcus* | 0 (0) | 12 (7·5) | 0 (0) | 11 (8·2) |
| *S. aureus* | 0 (0) | 14 (8·8) | 0 (0) | 9 (6·7) |
| *Streptococcus* | 0 (0) | 25 (15·6) | 0 (0) | 16 (11·9) |
| Other | 0 (0) | 31 (19·4) | 0 (0) | 28 (20·9) |
| Mixed | 0 (0) | 14 (8·8) | 0 (0) | 8 (6·0) |
| Viral | 0 (0) | 11 (6·9) | 0 (0) | 14 (10·4) |
| Non-infectious | 0 (0) | 33 (20·6) | 0 (0) | 28 (20·9) |
| Demographics |  |  |  |  |
| Age, years, mean (SD) | 63 (13) | 59·6 (14·4) | 57 (20) | 61·2 (15·3) |
| Gender, male (n, %) | 10 (83) | 99 (61·9) | 4 (57) | 79 (59·0) |
| Source of infection |  |  |  |  |
| Respiratory, n (%) | 5 (41) | 46 (36·2) | 3 (42) | 44 (41·5) |
| Abdominal, n (%) | 1 (8) | 45 (35·4) | 1 (14) | 34 (32·1) |
| Cardiovascular, n (%) | 3 (25) | 12 (9·4) | 4 (57) | 6 (5·7) |
| Urinary, n (%) | 1 (8) | 18 (14·2) | 0 (0) | 17 (16·0) |
| CNS, n (%) | 1 (8) | 5 (3·9) | 0 (0) | 11 (10·4) |
| Skin, n (%) | 2 (16) | 9 (7·1) | 0 (0) | 12 (11·3) |
| Unknown, n (%) | 3 (25) | 10 (7·9) | 2 (28) | 12 (11·3) |
| Other, n (%) | 1 (8) | 10 (7·9) | 1 (14) | 9 (8·5) |
| Severity of disease <24h |  |  |  |  |
| APACHE IV Score, mean (SD) | 84 (29) | 86·7 (31·2) | 84 (24) | 87·6 (37·6) |
| APS, mean (SD) | 72 (24) | 74·5 (29·8) | 73 (24) | 74·2 (35·6) |
| mSOFA score, median [IQR]^A^ | 5 [4, 8] | 8·0 [6·0, 10·0] | 7 [6, 9] | 7·0 [5·0, 10·0] |
| Shock, n (%)^B^ | 5 (41) | 90 (56·2) | 3 (42) | 61 (45·5) |
| Mortality |  |  |  |  |
| ICU | 2 (16) | 33 (20·6) | 2 (28) | 24 (17·9) |
| 30 days | 1 (8) | 45 (28·1) | 2 (28) | 31 (23·1) |
| 90 days | 3 (25) | 59 (36·9) | 2 (28) | 36 (26·9) |
| 1 year | 6 (50) | 72 (45·0) | 3 (42) | 54 (40·3) |

Note.

Data presented as mean with standard deviation (SD), or median [interquartile range], or n (%).

^A^ mSOFA modified sequential organ failure assessment (excluding central nervous system component).

^B^ Shock was defined by the use of vasopressors (norepinephrine, epinephrine or dopamine) for hypotension in a norepinephrine-equivalent dose of more than 0·1 µg/kg/min.

# Table S8. Baseline characteristics and outcome of patients stratified for the *E. coli* classifier analysis

|  | Discovery | | Validation | |
| --- | --- | --- | --- | --- |
|  | Case | Control | Case | Control |
| n | 27 | 166 | 13 | 116 |
| BSI group |  |  |  |  |
| CoNS | 0 (0·0) | 15 (9·0) | 0 (0) | 4 (3·4) |
| *E. coli* | 27 (100·0) | 0 (0·0) | 13 (100) | 0 (0·0) |
| *Enterococcus* | 0 (0·0) | 13 (7·8) | 0 (0) | 10 (8·6) |
| *S. aureus* | 0 (0·0) | 14 (8·4) | 0 (0) | 9 (7·8) |
| *Streptococcus* | 0 (0·0) | 26 (15·7) | 0 (0) | 15 (12·9) |
| Other | 0 (0·0) | 34 (20·5) | 0 (0) | 25 (21·6) |
| Mixed | 0 (0·0) | 15 (9·0) | 0 (0) | 16 (13·8) |
| Viral | 0 (0·0) | 9 (5·4) | 0 (0) | 16 (13·8) |
| Non-infectious | 0 (0·0) | 40 (24·1) | 0 (0) | 21 (18·1) |
| Demographics |  |  |  |  |
| Age, years, mean (SD) | 61·1 (12·8) | 59·8 (15·1) | 64 (12) | 59·4 (15·1) |
| Gender, male (n, %) | 17 (63·0) | 101 (60·8) | 5 (38) | 72 (62·1) |
| Source of infection |  |  |  |  |
| Respiratory, n (%) | 6 (22·2) | 40 (31·7) | 4 (30) | 55 (57·9) |
| Abdominal, n (%) | 15 (55·6) | 28 (22·2) | 5 (38) | 28 (29·5) |
| Cardiovascular, n (%) | 2 (7·4) | 20 (15·9) | 0 (0) | 8 (8·4) |
| Urinary, n (%) | 4 (14·8) | 17 (13·5) | 3 (23) | 11 (11·6) |
| CNS, n (%) | 0 (0·0) | 10 (7·9) | 0 (0) | 9 (9·5) |
| Skin, n (%) | 3 (11·1) | 15 (11·9) | 2 (15) | 6 (6·3) |
| Unknown, n (%) | 2 (7·4) | 15 (11·9) | 1 (7) | 9 (9·5) |
| Other, n (%) | 0 (0·0) | 17 (13·5) | 0 (0) | 7 (7·4) |
| Severity of disease <24h |  |  |  |  |
| APACHE IV Score, mean (SD) | 100·1 (33·7) | 85·2 (31·8) | 115 (45) | 81·4 (33·0) |
| APS, mean (SD) | 88·5 (32·8) | 72·7 (29·7) | 97 (42) | 69·3 (31·3) |
| mSOFA score, median [IQR]^A^ | 9·0 [7·0, 11·0] | 8·0 [5·0, 10·0] | 10 [5, 13] | 7·0 [5·0, 9·0] |
| Shock, n (%)^B^ | 20 (74·1) | 78 (47·0) | 8 (61) | 52 (44·8) |
| Mortality |  |  |  |  |
| ICU | 4 (14·8) | 33 (19·9) | 5 (38) | 22 (19·0) |
| 30 days | 7 (25·9) | 43 (25·9) | 5 (38) | 26 (22·4) |
| 90 days | 10 (37·0) | 57 (34·3) | 5 (38) | 32 (27·6) |
| 1 year | 12 (44·4) | 72 (43·4) | 5 (38) | 52 (44·8) |

Note.

Data presented as mean with standard deviation (SD), or median [interquartile range], or n (%).

^A^ mSOFA modified sequential organ failure assessment (excluding central nervous system component).

^B^ Shock was defined by the use of vasopressors (norepinephrine, epinephrine or dopamine) for hypotension in a norepinephrine-equivalent dose of more than 0·1 µg/kg/min.

# Table S9. Baseline characteristics and outcome of patients stratified for the *Enterococcus* classifier analysis

|  | Discovery | | Validation | |
| --- | --- | --- | --- | --- |
|  | Case | Control | Case | Control |
| n | 14 | 171 | 9 | 127 |
| BSI group |  |  |  |  |
| CoNS | 0 (0) | 7 (4·1) | 0 (0) | 12 (9·4) |
| *E. coli* | 0 (0) | 26 (15·2) | 0 (0) | 14 (11·0) |
| *Enterococcus* | 14 (100) | 0 (0·0) | 9 (100) | 0 (0·0) |
| *S. aureus* | 0 (0) | 14 (8·2) | 0 (0) | 9 (7·1) |
| *Streptococcus* | 0 (0) | 23 (13·5) | 0 (0) | 18 (14·2) |
| Other | 0 (0) | 34 (19·9) | 0 (0) | 24 (18·9) |
| Mixed | 0 (0) | 14 (8·2) | 0 (0) | 17 (13·4) |
| Viral | 0 (0) | 13 (7·6) | 0 (0) | 12 (9·4) |
| Non-infectious | 0 (0) | 40 (23·4) | 0 (0) | 21 (16·5) |
| Demographics |  |  |  |  |
| Age, years, mean (SD) | 62 (10) | 59·2 (14·9) | 63 (12) | 60·5 (15·2) |
| Gender, male (n, %) | 7 (50) | 104 (60·8) | 4 (44) | 84 (66·1) |
| Source of infection |  |  |  |  |
| Respiratory, n (%) | 5 (35) | 56 (42·7) | 1 (11) | 45 (42·5) |
| Abdominal, n (%) | 6 (42) | 42 (32·1) | 7 (77) | 18 (17·0) |
| Cardiovascular, n (%) | 1 (7) | 10 (7·6) | 2 (22) | 14 (13·2) |
| Urinary, n (%) | 4 (28) | 14 (10·7) | 2 (22) | 18 (17·0) |
| CNS, n (%) | 0 (0) | 7 (5·3) | 0 (0) | 12 (11·3) |
| Skin, n (%) | 0 (0) | 13 (9·9) | 0 (0) | 13 (12·3) |
| Unknown, n (%) | 3 (21) | 12 (9·2) | 0 (0) | 12 (11·3) |
| Other, n (%) | 1 (7) | 9 (6·9) | 0 (0) | 11 (10·4) |
| Severity of disease <24h |  |  |  |  |
| APACHE IV Score, mean (SD) | 90 (22) | 84·2 (31·0) | 79 (21) | 87·6 (38·0) |
| APS, mean (SD) | 74 (19) | 72·7 (29·7) | 65 (20) | 74·4 (35·4) |
| mSOFA score, median [IQR]^A^ | 9 [7, 11] | 8·0 [6·0, 10·0] | 7 [6, 10] | 7·0 [5·0, 10·0] |
| Shock, n (%)^B^ | 7 (50) | 86 (50·3) | 6 (66) | 54 (42·5) |
| Mortality |  |  |  |  |
| ICU | 7 (50) | 29 (17·0) | 2 (22) | 24 (18·9) |
| 30 days | 10 (71) | 37 (21·6) | 3 (33) | 29 (22·8) |
| 90 days | 11 (78) | 52 (30·4) | 3 (33) | 35 (27·6) |
| 1 year | 12 (85) | 71 (41·5) | 5 (55) | 48 (37·8) |

Note.

Data presented as mean with standard deviation (SD), or median [interquartile range], or n (%).

^A^ mSOFA modified sequential organ failure assessment (excluding central nervous system component).

^B^ Shock was defined by the use of vasopressors (norepinephrine, epinephrine or dopamine) for hypotension in a norepinephrine-equivalent dose of more than 0·1 µg/kg/min.

# Table S10. Baseline characteristics and outcome of patients stratified for the *S. aureus* classifier analysis

|  | Discovery | | Validation | |
| --- | --- | --- | --- | --- |
|  | Case | Control | Case | Control |
| n | 14 | 171 | 9 | 119 |
| BSI group |  |  |  |  |
| CoNS | 0 (0) | 15 (8·8) | 0 (0) | 4 (3·4) |
| *E. coli* | 0 (0) | 25 (14·6) | 0 (0) | 15 (12·6) |
| *Enterococcus* | 0 (0) | 12 (7·0) | 0 (0) | 11 (9·2) |
| *S. aureus* | 14 (100) | 0 (0·0) | 9 (100) | 0 (0·0) |
| *Streptococcus* | 0 (0) | 26 (15·2) | 0 (0) | 15 (12·6) |
| Other | 0 (0) | 30 (17·5) | 0 (0) | 29 (24·4) |
| Mixed | 0 (0) | 16 (9·4) | 0 (0) | 6 (5·0) |
| Viral | 0 (0) | 8 (4·7) | 0 (0) | 17 (14·3) |
| Non-infectious | 0 (0) | 39 (22·8) | 0 (0) | 22 (18·5) |
| Demographics |  |  |  |  |
| Age, years, mean (SD) | 61 (10) | 60·5 (15·1) | 62 (14) | 59·9 (15·1) |
| Gender, male (n, %) | 8 (57) | 108 (63·2) | 6 (66) | 70 (58·8) |
| Source of infection |  |  |  |  |
| Respiratory, n (%) | 2 (14) | 39 (22·8) | 6 (66) | 51 (42·9) |
| Abdominal, n (%) | 1 (7) | 44 (33·3) | 0 (0) | 36 (37·1) |
| Cardiovascular, n (%) | 4 (28) | 14 (10·6) | 3 (33) | 4 (4·1) |
| Urinary, n (%) | 2 (14) | 20 (15·2) | 1 (11) | 13 (13·4) |
| CNS, n (%) | 1 (7) | 8 (6·1) | 2 (22) | 6 (6·2) |
| Skin, n (%) | 4 (28) | 13 (9·8) | 2 (22) | 4 (4·1) |
| Unknown, n (%) | 3 (21) | 14 (10·6) | 0 (0) | 10 (10·3) |
| Other, n (%) | 4 (28) | 10 (7·6) | 3 (33) | 4 (4·1) |
| Severity of disease <24h |  |  |  |  |
| APACHE IV Score, mean (SD) | 98 (25) | 87·4 (33·5) | 99 (51) | 83·9 (33·4) |
| APS, mean (SD) | 86 (25) | 74·9 (31·7) | 84 (50) | 71·2 (31·4) |
| mSOFA score, median [IQR]^A^ | 8 [6, 10] | 8·0 [6·0, 10·0] | 9 [8, 12] | 7·0 [5·0, 10·0] |
| Shock, n (%)^B^ | 6 (42) | 94 (55·0) | 6 (66) | 53 (44·5) |
| Mortality |  |  |  |  |
| ICU | 4 (28) | 32 (18·7) | 1 (11) | 24 (20·2) |
| 30 days | 7 (50) | 42 (24·6) | 1 (11) | 29 (24·4) |
| 90 days | 7 (50) | 58 (33·9) | 3 (33) | 32 (26·9) |
| 1 year | 8 (57) | 72 (42·1) | 7 (77) | 48 (40·3) |

Note.

Data presented as mean with standard deviation (SD), or median [interquartile range], or n (%).

^A^ mSOFA modified sequential organ failure assessment (excluding central nervous system component).

^B^ Shock was defined by the use of vasopressors (norepinephrine, epinephrine or dopamine) for hypotension in a norepinephrine-equivalent dose of more than 0·1 µg/kg/min.

# Table S11. Baseline characteristics and outcome of patients stratified for the *Streptococcus* classifier analysis

|  | Discovery | | Validation | |
| --- | --- | --- | --- | --- |
|  | Case | Control | Case | Control |
| n | 27 | 170 | 14 | 120 |
| BSI group |  |  |  |  |
| CoNS | 0 (0·0) | 15 (8·8) | 0 (0) | 4 (3·3) |
| *E. coli* | 0 (0·0) | 22 (12·9) | 0 (0) | 18 (15·0) |
| *Enterococcus* | 0 (0·0) | 12 (7·1) | 0 (0) | 11 (9·2) |
| *S. aureus* | 0 (0·0) | 17 (10·0) | 0 (0) | 6 (5·0) |
| *Streptococcus* | 27 (100·0) | 0 (0·0) | 14 (100) | 0 (0·0) |
| Other | 0 (0·0) | 31 (18·2) | 0 (0) | 28 (23·3) |
| Mixed | 0 (0·0) | 26 (15·3) | 0 (0) | 14 (11·7) |
| Viral | 0 (0·0) | 11 (6·5) | 0 (0) | 14 (11·7) |
| Non-infectious | 0 (0·0) | 36 (21·2) | 0 (0) | 25 (20·8) |
| Demographics |  |  |  |  |
| Age, years, mean (SD) | 61·2 (15·2) | 59·5 (14·7) | 57 (14) | 61·4 (15·1) |
| Gender, male (n, %) | 18 (66·7) | 107 (62·9) | 8 (57) | 71 (59·2) |
| Source of infection |  |  |  |  |
| Respiratory, n (%) | 11 (40·7) | 42 (31·3) | 7 (50) | 46 (48·4) |
| Abdominal, n (%) | 2 (7·4) | 44 (32·8) | 1 (7) | 36 (37·9) |
| Cardiovascular, n (%) | 1 (3·7) | 20 (14·9) | 0 (0) | 8 (8·4) |
| Urinary, n (%) | 0 (0·0) | 24 (17·9) | 0 (0) | 15 (15·8) |
| CNS, n (%) | 4 (14·8) | 5 (3·7) | 4 (28) | 5 (5·3) |
| Skin, n (%) | 6 (22·2) | 13 (9·7) | 2 (14) | 6 (6·3) |
| Unknown, n (%) | 1 (3·7) | 15 (11·2) | 2 (14) | 9 (9·5) |
| Other, n (%) | 4 (14·8) | 11 (8·2) | 3 (21) | 6 (6·3) |
| Severity of disease <24h |  |  |  |  |
| APACHE IV Score, mean (SD) | 81·3 (25·0) | 86·0 (31·1) | 80 (28) | 86·9 (37·3) |
| APS, mean (SD) | 66·5 (24·2) | 74·2 (28·9) | 70 (28) | 73·6 (35·3) |
| mSOFA score, median [IQR]^A^ | 8·0 [5·0, 10·0] | 8·0 [6·0, 10·0] | 7 [6, 7] | 8·0 [5·0, 10·0] |
| Shock, n (%)^B^ | 12 (44·4) | 88 (51·8) | 8 (57) | 55 (45·8) |
| Mortality |  |  |  |  |
| ICU | 4 (14·8) | 32 (18·8) | 2 (14) | 27 (22·5) |
| 30 days | 6 (22·2) | 43 (25·3) | 3 (21) | 30 (25·0) |
| 90 days | 8 (29·6) | 60 (35·3) | 3 (21) | 35 (29·2) |
| 1 year | 8 (29·6) | 78 (45·9) | 5 (35) | 54 (45·0) |

Note.

Data presented as mean with standard deviation (SD), or median [interquartile range], or n (%).

^A^ mSOFA modified sequential organ failure assessment (excluding central nervous system component).

^B^ Shock was defined by the use of vasopressors (norepinephrine, epinephrine or dopamine) for hypotension in a norepinephrine-equivalent dose of more than 0·1 µg/kg/min.

# Table S12: Additional details and performance statistics of the STR8G classifier

| **a)** |  |
| --- | --- |
| **Gene** | **Beta coefficient** |
| *SORT1* | 1·37 |
| *CWF19L1* | 1·23 |
| *SEC24A* | 1·18 |
| *GALNT2* | 0·96 |
| *PLEKHO1* | -0·99 |
| *RASA3* | -1·03 |
| *ZNF175* | -1·03 |
| *ZNF589* | -0·98 |
|  |  |
| **Threshold** | 0·40 |

Note: The beta coefficients of the STR8G classifier were estimated from the univariate logistic tandem regression algorithm (see supplementary methods above)

**b)**

|  | **Discovery** | **Validation** | **Discovery** | **Validation** |
| --- | --- | --- | --- | --- |
|  | **(all)** | **(all)** | **(day 0 only)** | **(day 0 only)** |
| Sensitivity (recall) | 0·85 | 0·86 | 0·89 | 0·88 |
|  | (0.70, 0.97) | (0.64, 1.00) | (0.72, 1.00) | (0.60, 1.00) |
| Specificity | 0·76 | 0·71 | 0·76 | 0·71 |
|  | (0.69, 0.82) | (0.62, 0.78) | (0.69, 0.83) | (0.61, 0.80) |
| Positive predictive value (precision) | 0·36 | 0·26 | 0·34 | 0·22 |
|  | (0.25, 0.47) | (0.14, 0.38) | (0.20, 0.48) | (0.09, 0.37) |
| Negative predictive value | 0·97 | 0·98 | 0·98 | 0·98 |
|  | (0.94, 0.99) | (0.94, 1.00) | (0.95, 1.00) | (0.95, 1.00) |
| F1 | 0·51 | 0·39 | 0·49 | 0·35 |
|  | (0.38, 0.62) | (0.23, 0.54) | (0.32, 0.63) | (0.16, 0.53) |
| Prevalence | 0·14 | 0·10 | 0·12 | 0·09 |
|  | (0.09, 0.18) | (0.06, 0.16) | (0.07, 0.18) | (0.03, 0.15) |
| Detection Rate | 0·12 | 0·09 | 0·11 | 0·08 |
|  | (0.08, 0.16) | (0.04, 0.14) | (0.06, 0.16) | (0.02, 0.13) |
| Detection Prevalence | 0·325 | 0·351 | 0·322 | 0·344 |
|  | (0.26, 0.39) | (0.28, 0.43) | (0.25, 0.40) | (0.25, 0.44) |
| Balanced Accuracy | 0·805 | 0·783 | 0·823 | 0·790 |
|  | (0.72, 0.87) | (0.67, 0.87) | (0.73, 0.90) | (0.65, 0.89) |

Point estimates are shown along with 95% CIs (shown in parentheses) which are estimated from 1000 non-parametric bootstraps of the confusionMatrix function from caret. For each bootstrap iteration, we resampled the discovery data with replacement and recalculated the confusion matrix using the fixed optimal threshold of 0·40 and extracted each classification statistic (sensitivity, specificity, etc.) from each resampled confusion matrix. The confidence intervals were derived by taking the 2.5th and 97.5th percentiles of the bootstrap distributions for each performance metric.

# Table S13: Plasma host response biomarker pairwise comparisons (unadjusted)

1. **Cytokine release and systemic inflammatory responses**

| **Mean differences (biomarkers on natural log scale)** | | | | | | | | | | **P-values** | | | | | | |
| --- | --- | --- | --- | --- | --- | --- | --- | --- | --- | --- | --- | --- | --- | --- | --- | --- |
| **IL-6** | | | | | | | | | |  | | | | | | |
|  | CoNS | | EC | | ENT | | SA | | STR |  | CoNS | EC | ENT | SA | STR |  |
| NI (ref) | 0·38 | 2·08*** | | 1·15 | | 2·13** | | 1·96*** | |  | 0·59 | 0·0002 | 0·07 | 0·0023 | 0·0006 |  |
| CoNS (ref) |  | 1·70* | | 0·77 | | 1·75* | | 1·59* | |  |  | 0·018 | 0·31 | 0·037 | 0·032 |  |
| EC (ref) |  |  | | -0·93 | | 0·05 | | -0·12 | |  |  |  | 0·20 | 0·97 | 0·77 |  |
| ENT (ref) |  |  | |  | | 0·98 | | 0·82 | |  |  |  |  | 0·27 | 0·30 |  |
| SA (ref) |  |  | |  | |  | | -0·16 | |  |  |  |  |  | 0·83 |  |
|  |  |  | |  | |  | |  | |  |  |  |  |  |  |  |
| **IL-8** | | | | | | | | | |  | | | | | | |
|  | CoNS | EC | | ENT | | SA | | STR | |  |  |  |  |  |  |  |
| NI (ref) | 0·66 | 2·54*** | | 1·53** | | 1·56** | | 0·82 | |  | 0·22 | <0·0001 | 0·0056 | 0·0057 | 0·07 |  |
| CoNS (ref) |  | 1·88** | | 0·86 | | 0·90 | | 0·16 | |  |  | 0·0024 | 0·20 | 0·19 | 0·79 |  |
| EC (ref) |  |  | | -1·01 | | -0·98 | | -1·72** | |  |  |  | 0·10 | 0·12 | 0·0015 |  |
| ENT (ref) |  |  | |  | | 0·03 | | -0·70 | |  |  |  |  | 0·96 | 0·24 |  |
| SA (ref) |  |  | |  | |  | | -0·74 | |  |  |  |  |  | 0·23 |  |
|  |  |  | |  | |  | |  | |  |  |  |  |  |  |  |
| **IL-10** | | | | | | | | | | | | | | | | |
|  | CoNS | EC | | ENT | | SA | | STR | |  |  |  |  |  |  |  |
| NI (ref) | 0·21 | 1·77*** | | 0·99* | | 1·12* | | 0·85* | |  | 0·64 | <0·0001 | 0·032 | 0·018 | 0·028 |  |
| CoNS (ref) |  | 1·55** | | 0·78 | | 0·91 | | 0·64 | |  |  | 0·0029 | 0·17 | 0·12 | 0·21 |  |
| EC (ref) |  |  | | -0·78 | | -0·64 | | -0·91* | |  |  |  | 0·13 | 0·22 | 0·044 |  |
| ENT (ref) |  |  | |  | | 0·14 | | -0·14 | |  |  |  |  | 0·82 | 0·79 |  |
| SA (ref) |  |  | |  | |  | | -0·27 | |  |  |  |  |  | 0·60 |  |
|  |  |  | |  | |  | |  | |  |  |  |  |  |  |  |
| **IL-1ra** | | | | | | | | | | | | | | | | |
|  | CoNS | EC | | ENT | | SA | | STR | |  |  |  |  |  |  |  |
| NI (ref) | 0·20 | 1·77*** | | 1·24** | | 1·57** | | 1·21** | |  | 0·66 | <0·0001 | 0·0076 | 0·0011 | 0·0020 |  |
| CoNS (ref) |  | 1·57** | | 1·04 | | 1·37* | | 1·01* | |  |  | 0·0027 | 0·07 | 0·019 | 0·049 |  |
| EC (ref) |  |  | | -0·53 | | -0·20 | | -0·56 | |  |  |  | 0·31 | 0·71 | 0·22 |  |
| ENT (ref) |  |  | |  | | 0·33 | | -0·03 | |  |  |  |  | 0·57 | 0·95 |  |
| SA (ref) |  |  | |  | |  | | -0·36 | |  |  |  |  |  | 0·49 |  |
|  |  |  | |  | |  | |  | |  |  |  |  |  |  |  |
| **PCT** | | | | | | | | | | | | | | | | |
|  | CoNS | EC | | ENT | | SA | | STR | |  |  |  |  |  |  |  |
| NI (ref) | 0·57 | 2·70*** | | 0·97* | | 2·45*** | | 2·73*** | |  | 0·15 | <0·0001 | 0·02 | <0·0001 | <0·0001 |  |
| CoNS (ref) |  | 2·13*** | | 0·40 | | 1·88** | | 2·16*** | |  |  | 0·0001 | 0·46 | 0·002 | 0·0002 |  |
| EC (ref) |  |  | | -1·73** | | -0·25 | | 0·03 | |  |  |  | 0·0021 | 0·69 | 0·92 |  |
| ENT (ref) |  |  | |  | | 1·48* | | 1·76** | |  |  |  |  | 0·016 | 0·0026 |  |
| SA (ref) |  |  | |  | |  | | 0·29 | |  |  |  |  |  | 0·75 |  |
|  |  |  | |  | |  | |  | |  |  |  |  |  |  |  |
| **MMP-8** | | | | | | | | | | | | | | | |  |
|  | CoNS | EC | | ENT | | SA | | STR | |  |  |  |  |  |  |  |
| NI (ref) | 0·78 | 1·93*** | | 1·29** | | 2·42*** | | 2·05*** | |  | 0·07 | <0·0001 | 0·0032 | <0·0001 | <0·0001 |  |
| CoNS (ref) |  | 1·15* | | 0·51 | | 1·63** | | 1·27** | |  |  | 0·019 | 0·34 | 0·0036 | 0·0089 |  |
| EC (ref) |  |  | | -0·64 | | 0·49 | | 0·12 | |  |  |  | 0·19 | 0·33 | 0·77 |  |
| ENT (ref) |  |  | |  | | 1·13* | | 0·76 | |  |  |  |  | 0·043 | 0·11 |  |
| SA (ref) |  |  | |  | |  | | -0·37 | |  |  |  |  |  | 0·47 |  |
|  |  |  | |  | |  | |  | |  |  |  |  |  |  |  |
| **sTREM-1** | | | | | | | | | | | | | | | |  |
|  | CoNS | EC | | ENT | | SA | | STR | |  |  |  |  |  |  |  |
| NI (ref) | -0·12 | 0·37 | | 0·50* | | 0·75** | | 0·12 | |  | 0·57 | 0·05 | 0·023 | 0·0010 | 0·52 |  |
| CoNS (ref) |  | 0·49* | | 0·63* | | 0·88** | | 0·24 | |  |  | 0·047 | 0·022 | 0·0018 | 0·32 |  |
| EC (ref) |  |  | | 0·14 | | 0·39 | | -0·25 | |  |  |  | 0·58 | 0·12 | 0·25 |  |
| ENT (ref) |  |  | |  | | 0·25 | | -0·39 | |  |  |  |  | 0·37 | 0·11 |  |
| SA (ref) |  |  | |  | |  | | -0·64* | |  |  |  |  |  | 0·012 |  |
|  |  |  | |  | |  | |  | |  |  |  |  |  |  |  |
| **Tenascin-C** | | | | | | | | | | | | | | | |  |
|  | CoNS | EC | | ENT | | SA | | STR | |  |  |  |  |  |  |  |
| NI (ref) | 0·22 | 0·39** | | 0·41* | | 0·65*** | | 0·51*** | |  | 0·17 | 0·0045 | 0·011 | 0·0001 | 0·0002 |  |
| CoNS (ref) |  | 0·17 | | 0·19 | | 0·43* | | 0·29 | |  |  | 0·34 | 0·33 | 0·03 | 0·10 |  |
| EC (ref) |  |  | | 0·02 | | 0·26 | | 0·12 | |  |  |  | 0·91 | 0·16 | 0·44 |  |
| ENT (ref) |  |  | |  | | 0·24 | | 0·10 | |  |  |  |  | 0·23 | 0·57 |  |
| SA (ref) |  |  | |  | |  | | -0·14 | |  |  |  |  |  | 0·45 |  |
|  |  |  | |  | |  | |  | |  |  |  |  |  |  |  |
| **sCD163** | | | | | | | | | | | | | | | |  |
|  | CoNS | EC | | ENT | | SA | | STR | |  |  |  |  |  |  |  |
| NI (ref) | 0.79** | 1·51*** | | 0·94** | | 1·93*** | | 0·73** | |  | 0·006 | <0·0001 | 0·0012 | <0·0001 | 0·003 |  |
| CoNS (ref) |  | 0·72* | | 0·14 | | 1·14** | | -0·06 | |  |  | 0·024 | 0·68 | 0·0017 | 0·84 |  |
| EC (ref) |  |  | | -0·58 | | 0·42 | | -0·78** | |  |  |  | 0·07 | 0·20 | 0·0055 |  |
| ENT (ref) |  |  | |  | | 0·99** | | -0·21 | |  |  |  |  | 0·006 | 0·51 |  |
| SA (ref) |  |  | |  | |  | | -1·20*** | |  |  |  |  |  | 0·0003 |  |
|  | | | | | | | | | |  | | | | | | |

1. **Endothelial cell and procoagulant responses**

| **Mean differences (biomarkers on natural log scale)** | | | | | | **P-values** | | | | | |  |
| --- | --- | --- | --- | --- | --- | --- | --- | --- | --- | --- | --- | --- |
| **Fractalkine** | | | | | |  | | | | | |  |
|  | CoNS | EC | ENT | SA | STR |  | CoNS | EC | ENT | SA | STR | |
| NI (ref) | 0.44 | 1.29*** | 0.99*** | 1.35*** | 0.66** |  | 0·11 | <0·0001 | 0·0004 | <0·0001 | 0·0051 | |
| CoNS (ref) |  | 0.85** | 0.56 | 0.92 | 0.22 |  |  | 0·0048 | 0·098 | 0·0082 | 0·46 | |
| EC (ref) |  |  | -0.30 | 0.06 | -0.63* |  |  |  | 0·32 | 0·84 | 0·017 | |
| ENT (ref) |  |  |  | 0.36 | -0.34 |  |  |  |  | 0·30 | 0·27 | |
| SA (ref) |  |  |  |  | -0.69* |  |  |  |  |  | 0·027 | |
|  |  |  |  |  |  |  |  |  |  |  |  | |
| **sThrombomodulin** | | | | | |  | | | | | |  |
|  | CoNS | EC | ENT | SA | STR |  |  |  |  |  |  | |
| NI (ref) | 0.06 | 0.30* | 0.41** | 0.61*** | 0.14 |  | 0·70 | 0·019 | 0·0076 | 0·0001 | 0·28 | |
| CoNS (ref) |  | 0.25 | 0.35 | 0.55** | 0.08 |  |  | 0·14 | 0·062 | 0·0040 | 0·64 | |
| EC (ref) |  |  | 0.10 | 0.31 | -0.17 |  |  |  | 0·54 | 0·077 | 0·25 | |
| ENT (ref) |  |  |  | 0.20 | -0.27 |  |  |  |  | 0·29 | 0·11 | |
| SA (ref) |  |  |  |  | -0.47** |  |  |  |  |  | 0·0062 | |
|  |  |  |  |  |  |  |  |  |  |  |  | |
| **Syndecan-1** | | | | | |  | | | | | |  |
|  | CoNS | EC | ENT | SA | STR |  |  |  |  |  |  | |
| NI (ref) | 0.18 | 0.63*** | 0.64** | 0.92*** | 0.17 |  | 0·40 | 0·0009 | 0·0036 | <0·0001 | 0·36 | |
| CoNS (ref) |  | 0.44 | 0.46 | 0.73** | -0.02 |  |  | 0·071 | 0·090 | 0·0084 | 0·95 | |
| EC (ref) |  |  | 0.02 | 0.29 | -0.46* |  |  |  | 0·95 | 0·25 | 0·033 | |
| ENT (ref) |  |  |  | 0.27 | -0.47 |  |  |  |  | 0·32 | 0·052 | |
| SA (ref) |  |  |  |  | -0.75** |  |  |  |  |  | 0·0030 | |
|  |  |  |  |  |  |  |  |  |  |  |  | |
| **Endocan** | | | | | |  | | | | | |  |
|  | CoNS | EC | ENT | SA | STR |  |  |  |  |  |  | |
| NI (ref) | -0.72 | 0.77* | 0.83* | 1.02* | -0.13 |  | 0·068 | 0·023 | 0·037 | 0·013 | 0·69 | |
| CoNS (ref) |  | 1.49*** | 1.55** | 1.74*** | 0.59 |  |  | 0·0009 | 0·0017 | 0·0006 | 0·18 | |
| EC (ref) |  |  | 0.06 | 0.25 | -0.90* |  |  |  | 0·89 | 0·58 | 0·021 | |
| ENT (ref) |  |  |  | 0.19 | -0.96* |  |  |  |  | 0·70 | 0·030 | |
| SA (ref) |  |  |  |  | -1.15* |  |  |  |  |  | 0·011 | |
|  |  |  |  |  |  |  |  |  |  |  |  | |
| **ANG-1** | | | | | |  | | | | | |  |
|  | CoNS | EC | ENT | SA | STR |  |  |  |  |  |  | |
| NI (ref) | -0.23 | -0.57** | 0.07 | -0.51* | -0.15 |  | 0·28 | 0·0025 | 0·74 | 0·023 | 0·41 | |
| CoNS (ref) |  | -0.33 | 0.31 | -0.27 | 0.08 |  |  | 0·17 | 0·25 | 0·31 | 0·73 | |
| EC (ref) |  |  | 0.64** | 0.06 | 0.42 |  |  |  | 0·0091 | 0·81 | 0·051 | |
| ENT (ref) |  |  |  | -0.58* | -0.22 |  |  |  |  | 0·034 | 0·35 | |
| SA (ref) |  |  |  |  | 0.36 |  |  |  |  |  | 0·15 | |
|  |  |  |  |  |  |  |  |  |  |  |  | |
| **ANG-2** | | | | | |  | | | | | |  |
|  | CoNS | EC | ENT | SA | STR |  |  |  |  |  |  | |
| NI (ref) | 0.19 | 1.08*** | 0.45* | 1.20*** | 0.94*** |  | 0·42 | <0·0001 | 0·049 | <0·0001 | <0·0001 | |
| CoNS (ref) |  | 0.90*** | 0.26 | 1.01*** | 0.75** |  |  | 0·0006 | 0·35 | 0·0005 | 0·0033 | |
| EC (ref) |  |  | -0.63* | 0.12 | -0.14 |  |  |  | 0·014 | 0·65 | 0·52 | |
| ENT (ref) |  |  |  | 0.75** | 0.49 |  |  |  |  | 0·0094 | 0·055 | |
| SA (ref) |  |  |  |  | -0.26 |  |  |  |  |  | 0·31 | |
|  |  |  |  |  |  |  |  |  |  |  |  | |
| **sTissue-Factor** | | | | | |  | | | | | |  |
|  | CoNS | EC | ENT | SA | STR |  |  |  |  |  |  | |
| NI (ref) | 0.03 | 0.18 | 0.54*** | 0.68*** | -0.01 |  | 0·83 | 0·16 | 0·0005 | <0·0001 | 0·93 | |
| CoNS (ref) |  | 0.15 | 0.51** | 0.65*** | -0.04 |  |  | 0·38 | 0·0072 | 0·0009 | 0·79 | |
| EC (ref) |  |  | 0.36* | 0.50** | -0.19 |  |  |  | 0·035 | 0·0048 | 0·19 | |
| ENT (ref) |  |  |  | 0.13 | -0.56** |  |  |  |  | 0·48 | 0·0012 | |
| SA (ref) |  |  |  |  | -0.69*** |  |  |  |  |  | <0·0001 | |
| **D-Dimer** | | | | | |  | | | | | |  |
|  | CoNS | EC | ENT | SA | STR |  |  |  |  |  |  | |
| NI (ref) | 0.16 | 0.25 | 0.02 | 0.13 | 0.13 |  | 0·40 | 0·12 | 0·92 | 0·49 | 0·41 | |
| CoNS (ref) |  | 0.09 | -0.14 | -0.03 | -0.03 |  |  | 0·65 | 0·55 | 0·91 | 0·90 | |
| EC (ref) |  |  | -0.23 | -0.12 | -0.12 |  |  |  | 0·27 | 0·57 | 0·51 | |
| ENT (ref) |  |  |  | 0.11 | 0.11 |  |  |  |  | 0·63 | 0·59 | |
| SA (ref) |  |  |  |  | 0.00 |  |  |  |  |  | 1·00 | |
|  |  |  |  |  |  |  |  |  |  |  |  | |
| **PT** | | | | | |  | | | | | |  |
|  | CoNS | EC | ENT | SA | STR |  |  |  |  |  |  | |
| NI (ref) | 0.15 | 0.21* | 0.16 | 0.27* | -0.06 |  | 0·25 | 0·049 | 0·21 | 0·041 | 0·57 | |
| CoNS (ref) |  | 0.07 | 0.01 | 0.12 | -0.21 |  |  | 0·64 | 0·95 | 0·44 | 0·14 | |
| EC (ref) |  |  | -0.06 | 0.05 | -0.28* |  |  |  | 0·69 | 0·70 | 0·030 | |
| ENT (ref) |  |  |  | 0.11 | -0.22 |  |  |  |  | 0·48 | 0·12 | |
| SA (ref) |  |  |  |  | -0.33* |  |  |  |  |  | 0·022 | |
|  |  |  |  |  |  |  |  |  |  |  |  | |
| **Platelets** | | | | | |  | | | | | |  |
|  | CoNS | EC | ENT | SA | STR |  |  |  |  |  |  | |
| NI (ref) | 0.20 | -0.31 | -0.15 | -0.24 | -0.09 |  | 0·37 | 0·096 | 0·49 | 0·28 | 0·63 | |
| CoNS (ref) |  | -0.51 | -0.35 | -0.44 | -0.29 |  |  | 0·040 | 0·20 | 0·11 | 0·24 | |
| EC (ref) |  |  | 0.16 | 0.07 | 0.22 |  |  |  | 0·51 | 0·79 | 0·32 | |
| ENT (ref) |  |  |  | -0.09 | 0.06 |  |  |  |  | 0·74 | 0·81 | |
| SA (ref) |  |  |  |  | 0.15 |  |  |  |  |  | 0·54 | |

Note.

Numbers represent mean differences across pairwise comparison, on natural log scale.

Red: denotes if the mean difference is higher compared to the reference BSI group.

Blue: denotes if the mean difference is lower compared to the reference BSI group.

Differences in (log-transformed) plasma biomarker distributions between BSI groups were compared using linear regression with contrast dummy coding. * p-value < 0.05, ** p-value < 0.01, *** p-value < 0.001

A robust sandwich estimator (HC3) was applied for Platelets due to heterogenous variances between groups.

ref: reference category; NI: non-infectious controls; CoNS: coagulase-negative staphylococci; EC: *E.coli*; ENT: *Enterocococcus*; SA: *S. aureus*; STR: *Streptococcus.*

ANG: angiopoietin, CD: cluster of differentiation, IL: interleukin, MMP: matrix metalloproteinase, PCT: procalcitonin, PT: prothrombin time, s: soluble, TREM, triggering receptor expressed on myeloid cells.


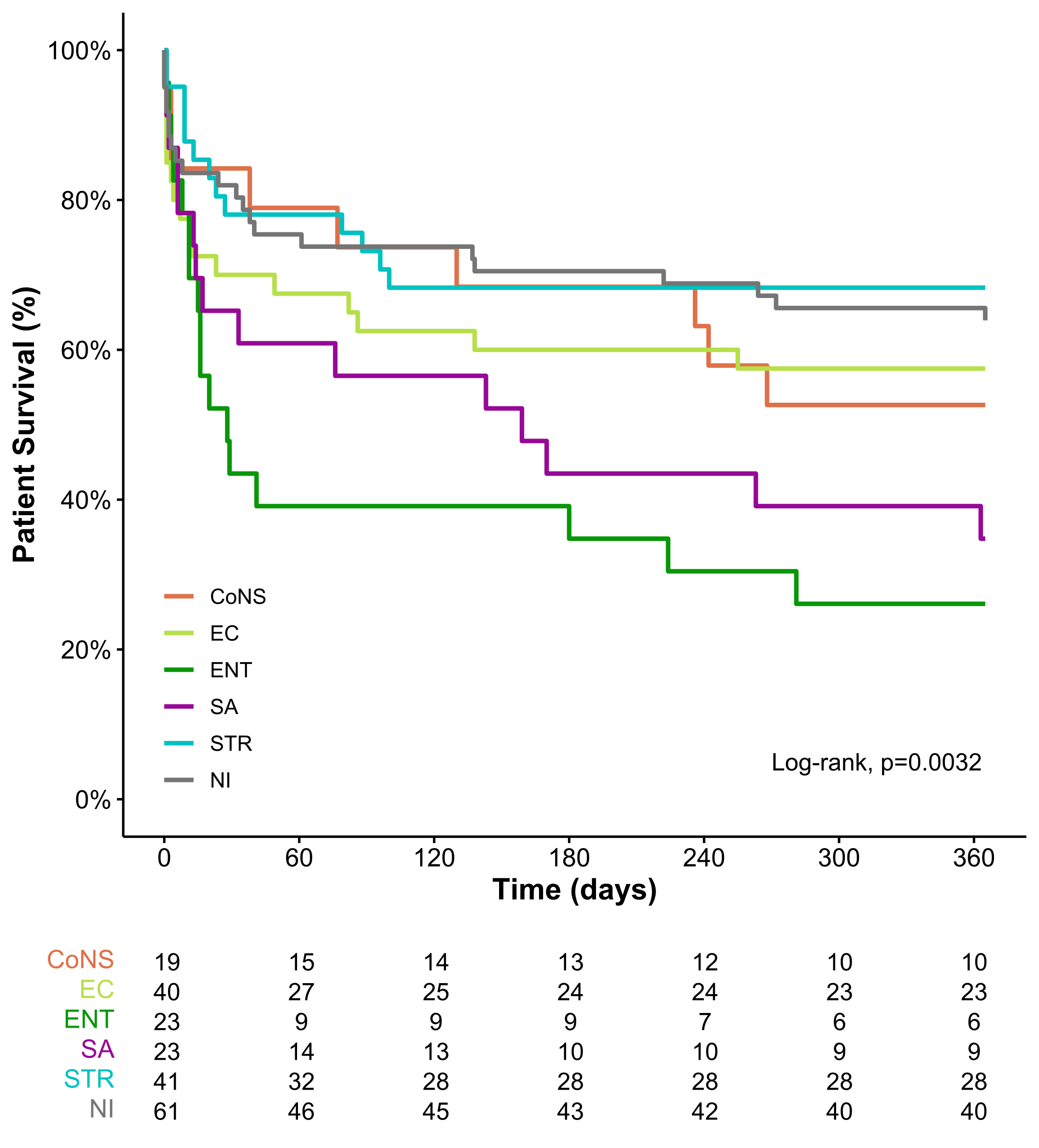

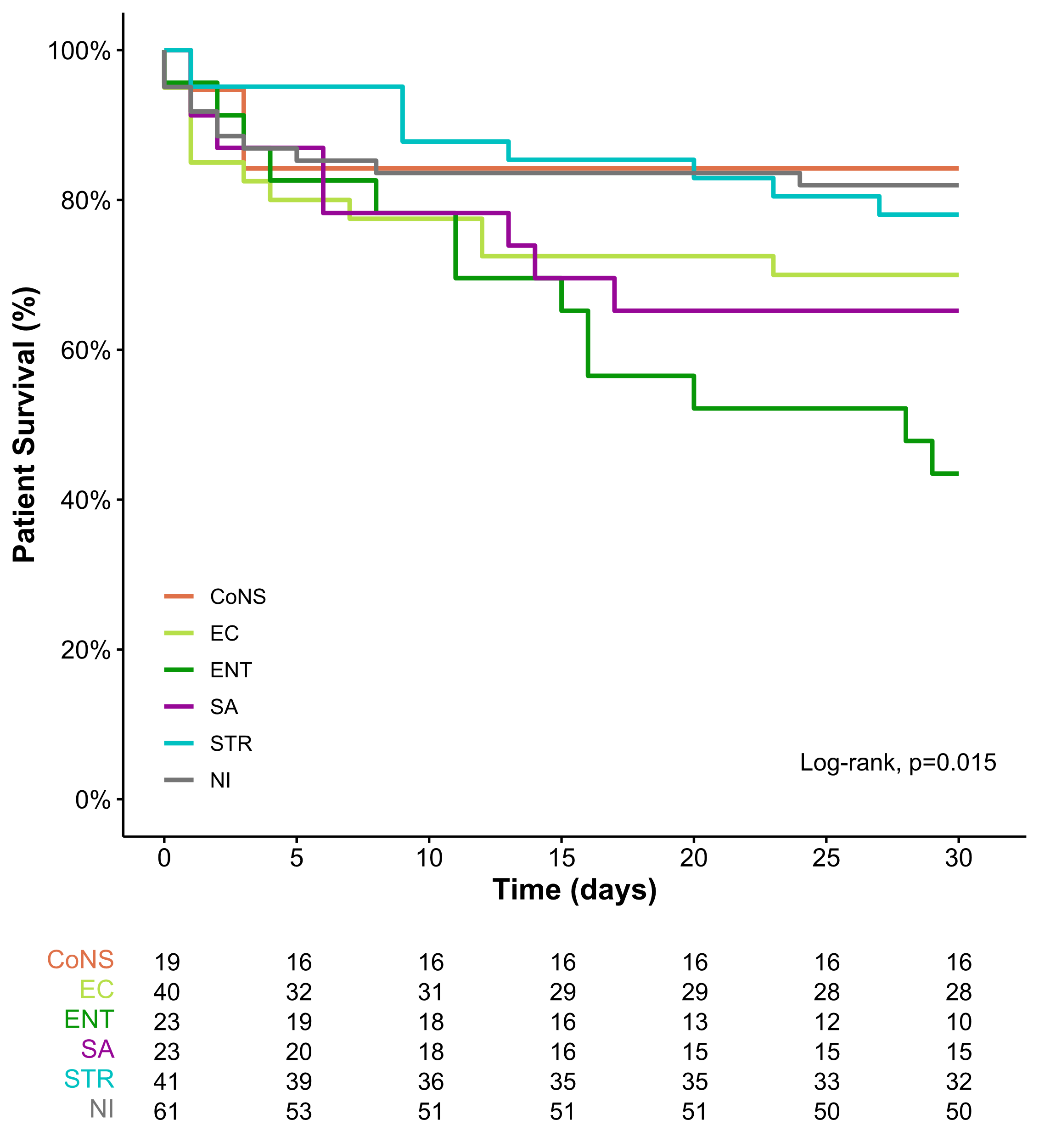


# Figure S1: Kaplan-Meier Survival Curves for BSI groups

The Kaplan-Meier plots depict the survival curves for the BSI groups over a follow-up period of 30 days (left), and one year (right). The log-rank test was used to compare the survival distributions between the groups, with both p-values indicating a significant difference in survival between the groups. The tables below the plots show the number of patients at risk in each group at the given time points. Up to one year, follow-up data of all patients was known.

CoNS: coagulase-negative staphylococci ; EC: *E. coli* ; ENT: *Enterocococcus* ; SA: *S. aureus* ; STR: *Streptococcus*; NI: non-infectious controls

| **Follow-up time (days) until one year** | | | | |
| --- | --- | --- | --- | --- |
| **BSI group** | **Median** | **IQR** | **Q1** | **Q3** |
| CoNS | 268 | 308 | 57 | 365 |
| EC | 365 | 353 | 12 | 365 |
| ENT | 28 | 312 | 11 | 323 |
| SA | 159 | 352 | 13 | 365 |
| STR | 365 | 286 | 79 | 365 |
| NI | 365 | 304 | 61 | 365 |


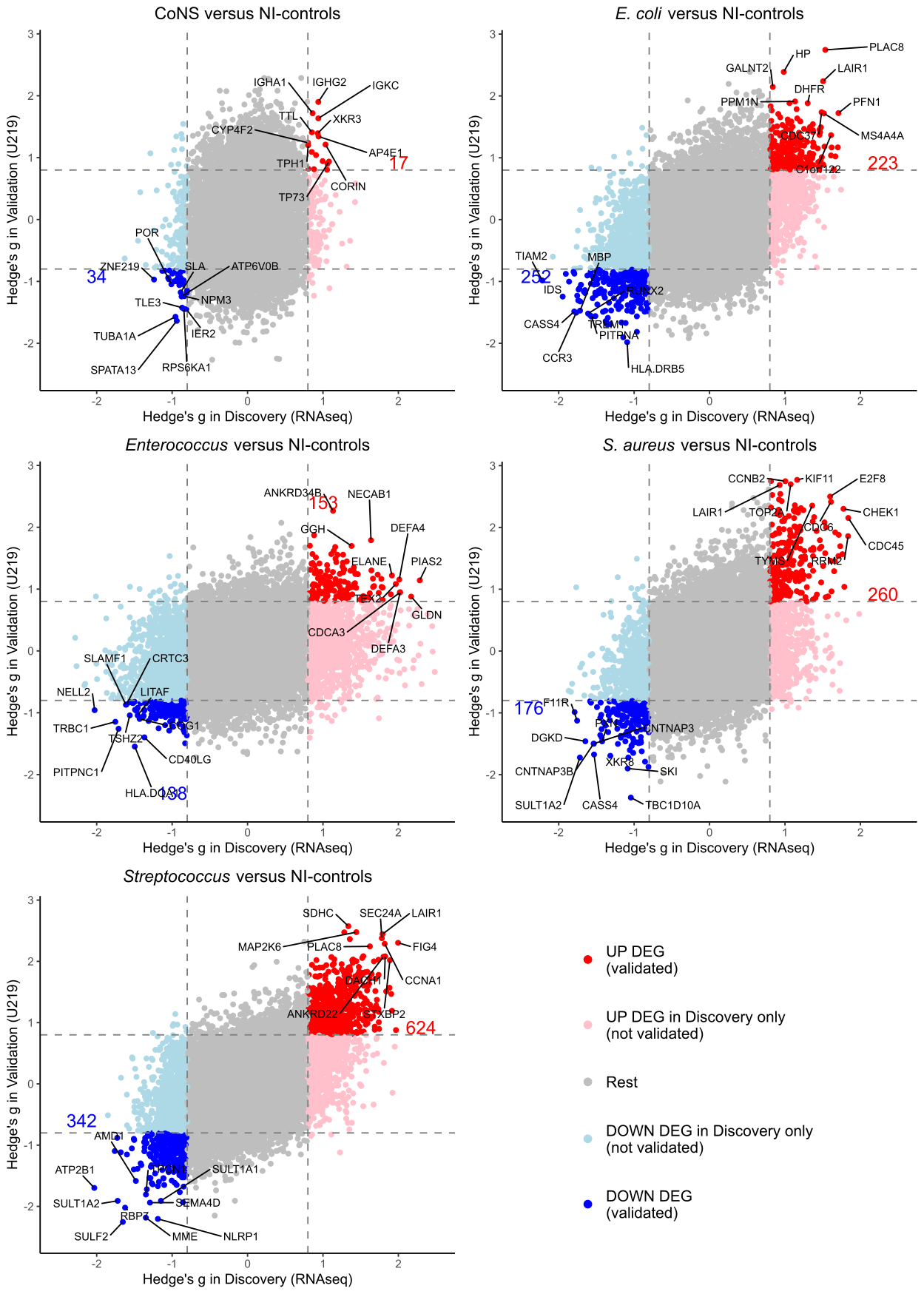


# Figure S2: Scatter plots showing the effect size of gene expression when comparing each bacterial group to NI-controls for all genes in two independent cohorts; discovery cohort (RNAseq) versus validation cohort (U219)

Upregulated DEGs are shown in red and down-regulated DEGs in blue, with the corresponding number of DEGs shown in the same colour. The highest 10 up and 10 down regulated DEGs are labelled by symbol. Genes that were a DEG in the discovery but were not validated are also highlighted, in pink and light blue.

**
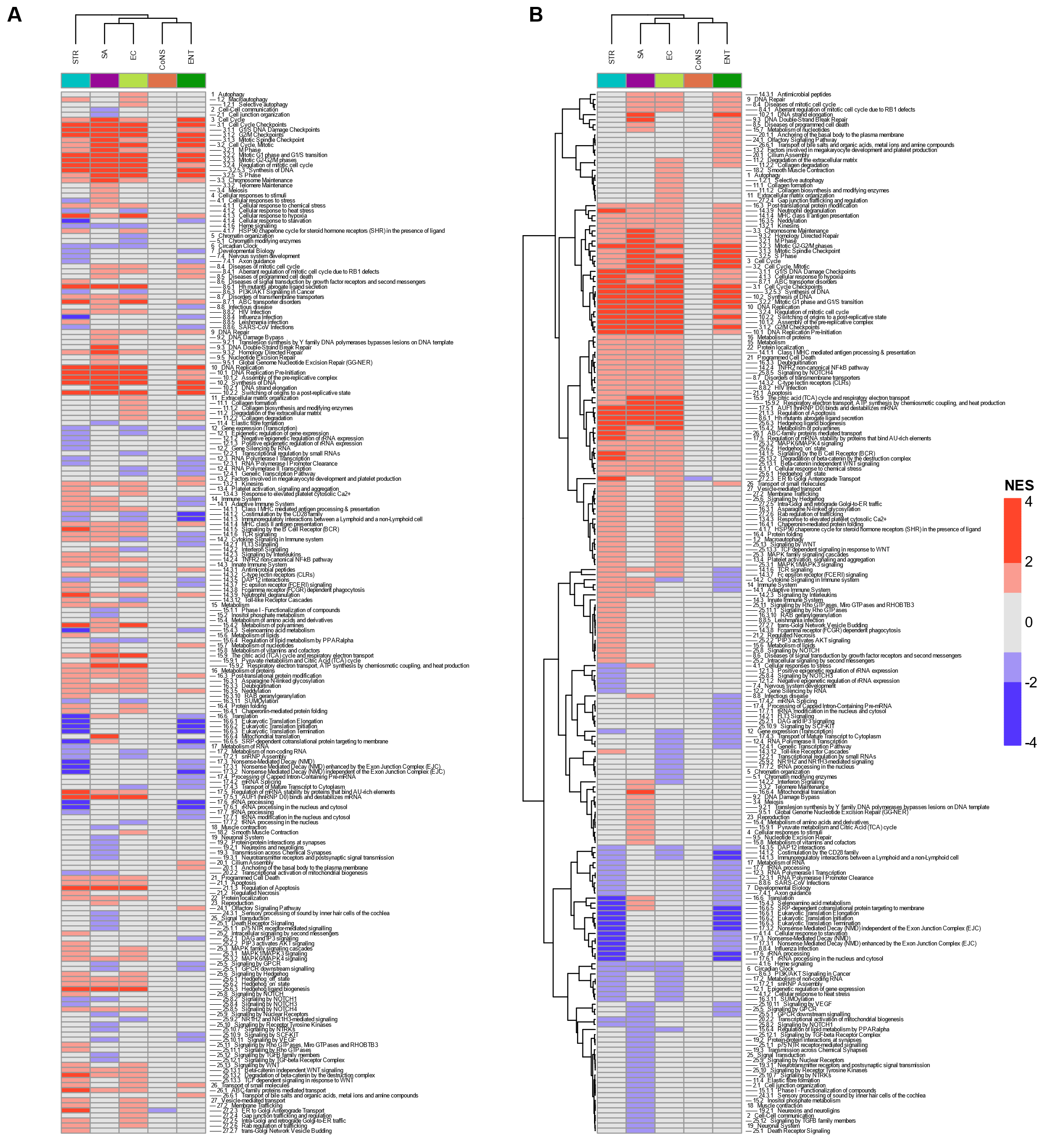
**

**b**

**a**

Figure S3: Heatmaps showing mean normalized enrichment scores (NES) from GSEA pathway analyses comparing each BSI group to non-infectious controls. Reactome pathways significant for at least one BSI group are shown, i·e· if BH-adjusted-P < 0·05 in both discovery and validation cohorts, and the direction of regulation was concordant between them (same sign of NES)· Here pathways from the three highest levels of the Reactome database (206 pathways) are displayed by **(a)** alphabetically ordering the nested pathway names, and by **(b)** hierarchical clustering based on the mean of the two NES scores from discovery (RNAseq) and validation (U219) cohorts. Heatmaps for the four highest levels of Reactome database (344 pathways) can be viewed on the following clickable links: [(a)](https://raw.githubusercontent.com/joemb1/PathwayHeatmaps/main/SuppGlobalHM_v_NI_order2.svg) and [(b)](https://raw.githubusercontent.com/joemb1/PathwayHeatmaps/main/SuppGlobalHM_v_NI_clust2.svg).

CoNS: coagulase-negative staphylococci; EC: *E. coli*; ENT: *Enterocococcus*; SA: *S. aureus* ; STR: *Streptococcus*

**
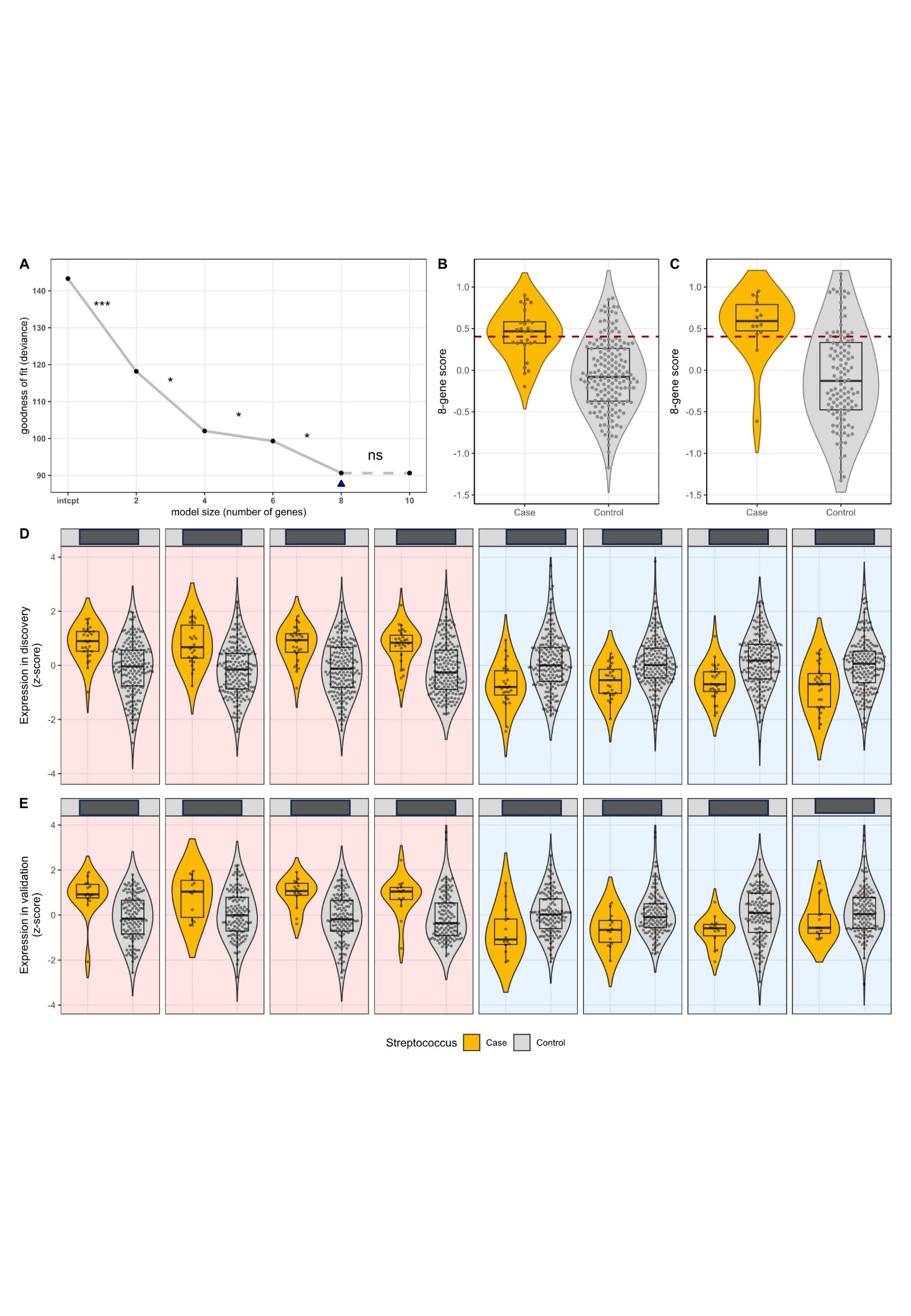
**

**e**

**d**

**b**

**c**

**a**

Figure S4: Selection and performance of an eight-gene classifier for *Streptococcus* BSIs (STR8G) **(a)** Showing goodness-of-fit of the ULTRA model in predicting case/control outcome with different number of predictors (genes). For model selection we began from a model with no genes (intercept model; intcpt) and iteratively added two predictors (the most significant up-regulated gene and down-regulated gene, after removing genes highly correlated with the existing added genes) until the improvement in the model fit was no longer significant· The selected model is indicated with a blue triangle, which is located at the end of the solid gray line· Significance was determined by a likelihood ratio test: *** P<0·001, * P<0·05, ns: not significant. **(b)** Violin plot showing distribution of the STR8G classifier score in the cases and controls of the discovery cohort. The red dashed line indicates the optimal threshold as defined by the Youden index of the discovery ROC (see Figure 4A). **(c)** Same for validation cohort. **(d)** Violin plots showing the distribution of expression of the individual genes that make up this score in cases and controls of the discovery cohort. **(e)** Same for the validation cohort.


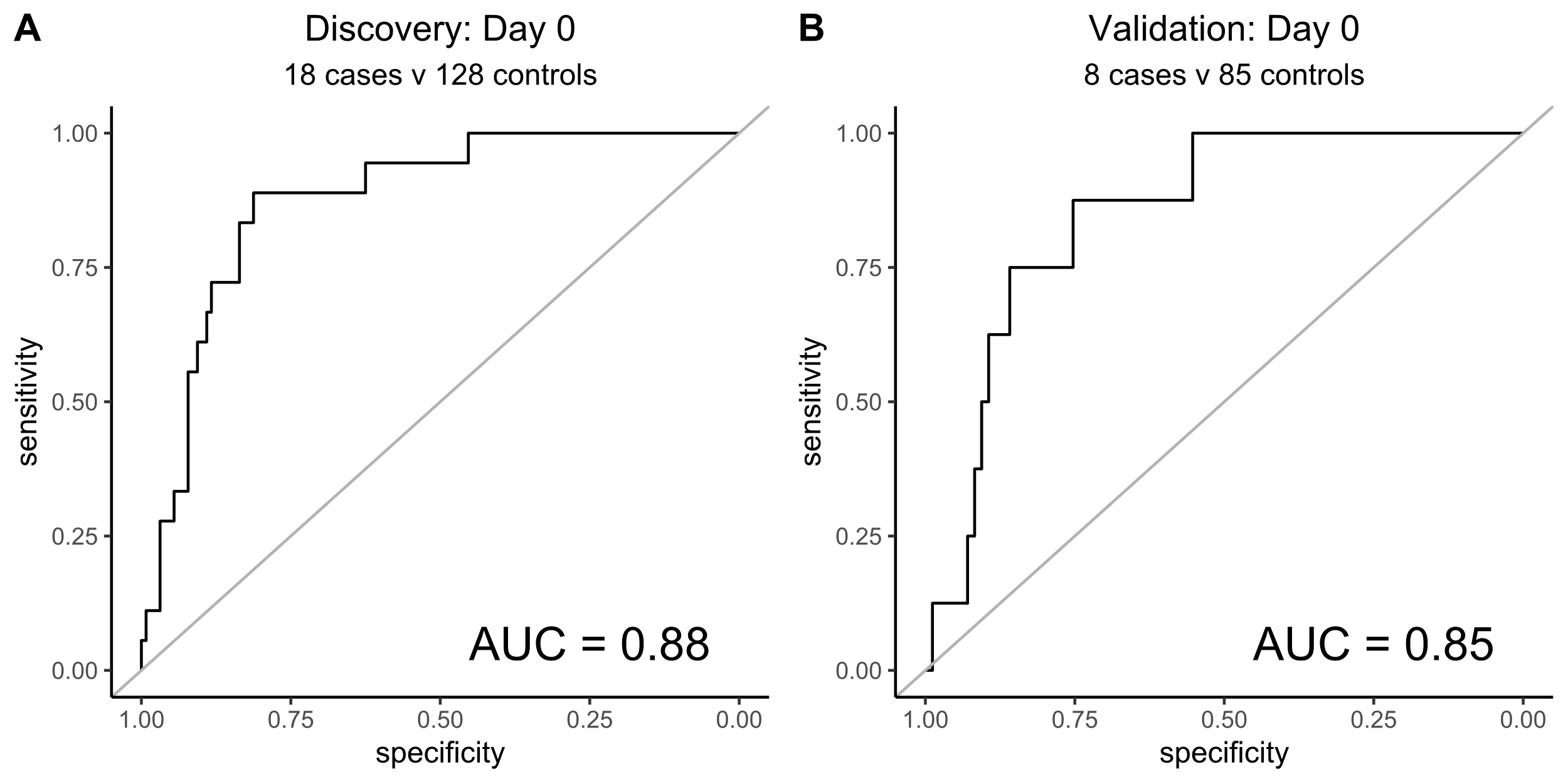


**b**

**a**

# Figure S5: STR8G classifier performance evaluated in patients with BC taken on same day as PAXgene blood draw.

(**a**) Discovery cohort. (**b**) Validation cohort.


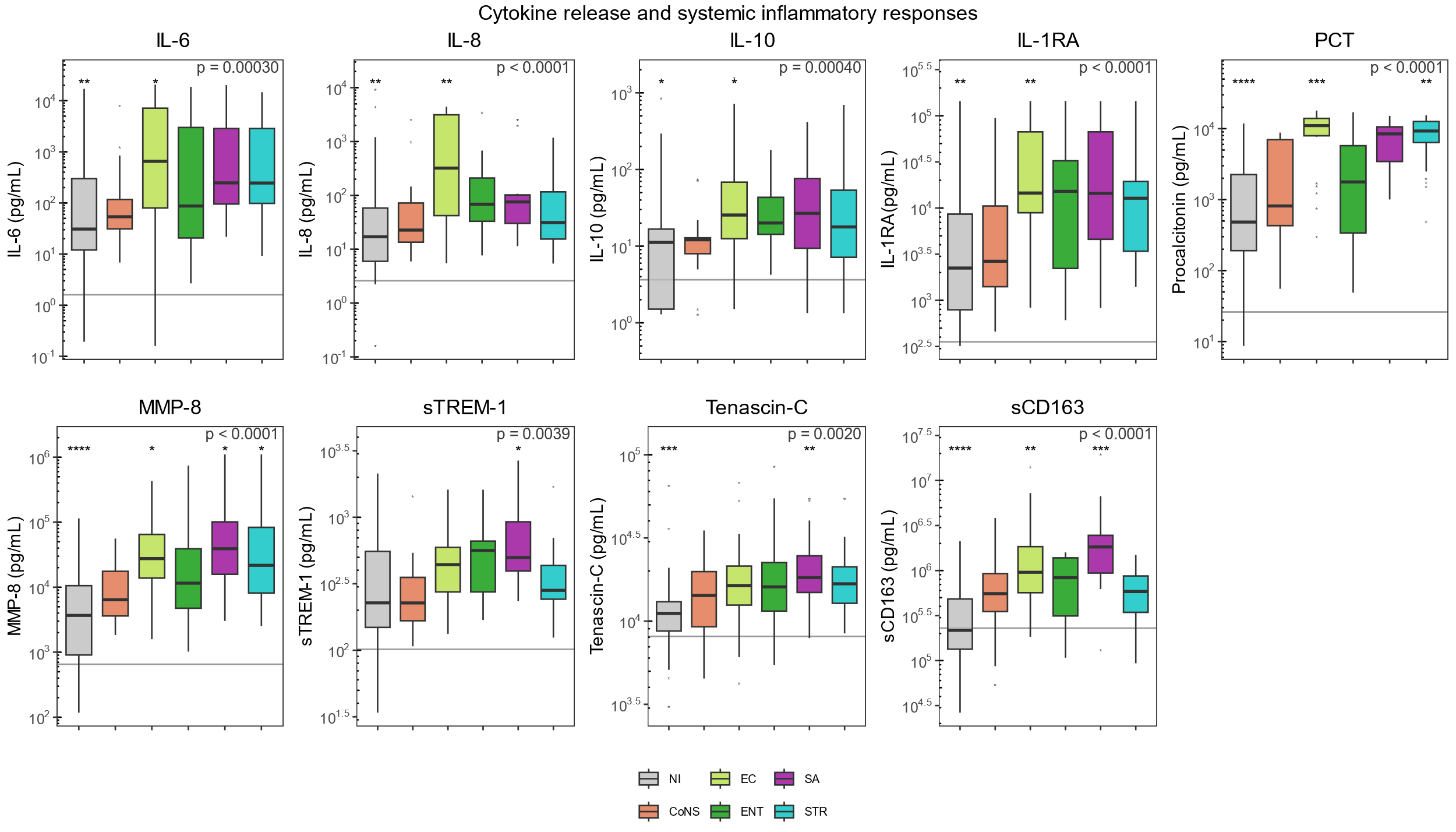


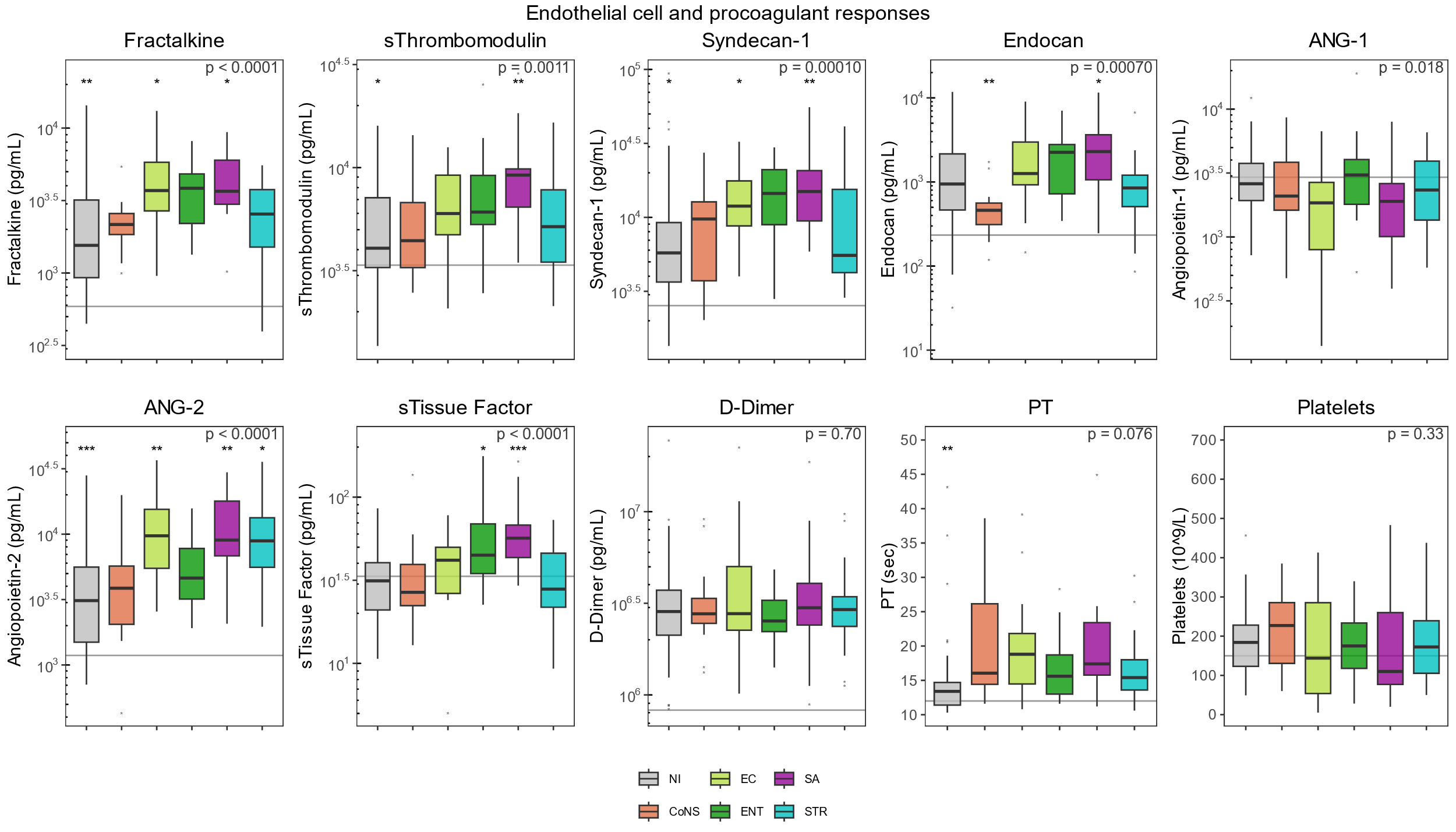


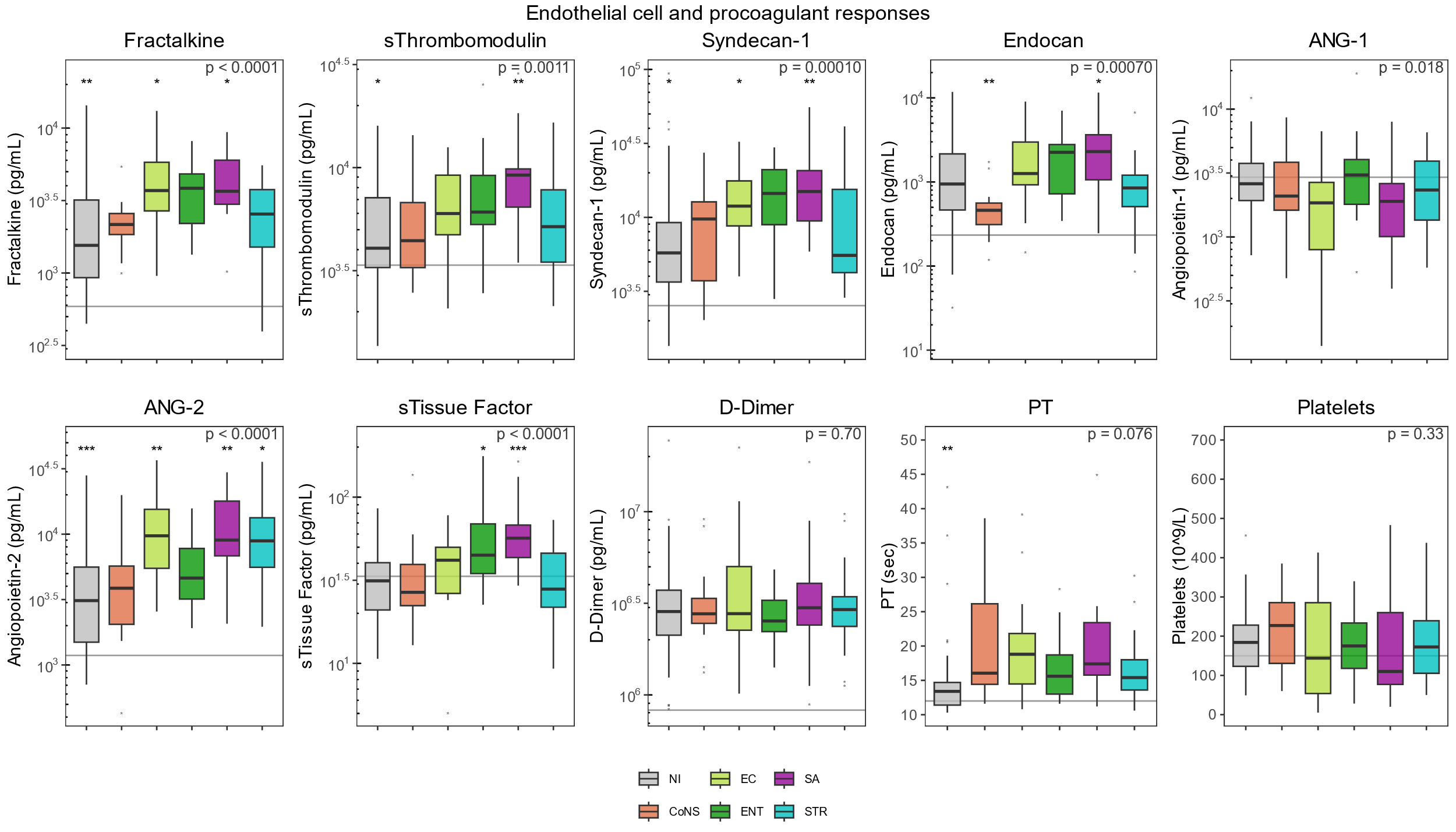


# Figure S6: Plasma host response biomarkers stratified by BSI group

Cytokine and inflammatory response markers (Top panel). Endothelial cell and procoagulant response markers (Bottom panel). Data are shown as box and whiskers. P-values in the top-right represent type-III Wald tests for the BSI group derived from linear regression models. Patients shown with the light gray points are outliers, defined as those falling more than 1.5 times the interquartile range (IQR) from the first or third quartile. Asterisks indicate differences between groups compared to the base-mean (*P < 0·05, **P < 0·01, ***P < 0·001, ****P < 0·0001). ANG: angiopoietin, CD: cluster of differentiation, IL: interleukin, MMP: matrix metalloproteinase, PCT: procalcitonin, PT: prothrombin time, s: soluble, TREM, triggering receptor expressed on myeloid cells.

CoNS: coagulase-negative staphylococci; EC: *E. coli*; ENT: *Enterocococcus*; SA: *S. aureus* ; STR: *Streptococcus*.

# References

1. Charlson, M.E Pompei, P. Ales, K.L. et al. A new method of classifying prognostic comorbidity in longitudinal studies: development and validation. *J Chronic Dis.* 1987;40(5):373–83.
2. Bernard, G.R. Artigas, A. Brigham, K.L. et al. The American-European Consensus Conference on ARDS. Definitions, mechanisms, relevant outcomes, and clinical trial coordination. *Am J Respir Crit Care Med.* 1994;149(3 Pt 1):818–24.
3. Khwaja, A. KDIGO clinical practice guidelines for acute kidney injury. *Nephron Clin Pract.* 2012;120(4).
4. Bellomo, R. Ronco, C. Kellum, J.A. et al. Acute renal failure - definition, outcome measures, animal models, fluid therapy and information technology needs: the Second International Consensus Conference of the Acute Dialysis Quality Initiative (ADQI) Group. *Crit Care.* 2004;8(4).
5. Bolger, A.M. Lohse, M. Usadel, B. Trimmomatic: A flexible trimmer for Illumina sequence data.
   *Bioinformatics.* 2014;30(15):2114–20.
6. Kim, D. Paggi, JM. Park, C. at al. Graph-based genome alignment and genotyping with HISAT2 and HISAT-genotype. *Nat Biotechnol.* 2019;37(8):907–15.
7. Anders, S. Pyl, P.T. Huber, W. HTSeq-A Python framework to work with high-throughput sequencing data.
   *Bioinformatics.* 2015;31(2):166–9.
8. Scicluna, B.P. Klein Klouwenberg, P.M.C. Van Vught, L.A. et al. A molecular biomarker to diagnose community-acquired pneumonia on intensive care unit admission. *Am J Respir Crit Care Med.* 2015;192(7):826–35.
9. Scicluna, B.P. Uhel, F. Van Vught, L.A. et al. The leukocyte non-coding RNA landscape in critically ill patients with sepsis. *Elife.* 2020;9:1–23.
10. Schuurman, A.R. Reijnders, T.D.Y. van Engelen, T.S.R. et al. The host response in different aetiologies of community-acquired pneumonia. *eBioMedicine.* 2022;81, 104059.
11. Reijnders, T.D.Y. Laterre, P.F. François, B. et al. Effect of mesenchymal stem cells on the host response in severe community-acquired pneumonia. *Thorax.* 2024;79(11).
12. van Engelen, T.S.R. Reijnders, T.D.Y. Paling, F.P. et al. Plasma protein biomarkers reflective of the host response in patients developing Intensive Care Unit-acquired pneumonia. *Crit Care.* 2023;27(1).
13. Hedges, L.V. Distribution Theory for Glass’s Estimator of Effect Size and Related Estimators. *J Educ Stat.* 1981;6(2):107.
